# Chimpanzees balance resources and risk in an anthropogenic landscape of fear

**DOI:** 10.1038/s41598-021-83852-3

**Published:** 2021-02-25

**Authors:** Elena Bersacola, Catherine M. Hill, Kimberley J. Hockings

**Affiliations:** 1 Centre for Ecology and Conservation, University of Exeter, Penryn, Cornwall, TR10 9FE UK; 2 Department of Social Sciences, Oxford Brookes University, Oxford, UK; 3 Centre for Research in Anthropology (CRIA), Lisbon, Portugal

**Keywords:** Ecology, Community ecology, Conservation biology, Ecological modelling

## Abstract

Human-wildlife coexistence is possible when animals can meet their ecological requirements while managing human-induced risks. Understanding how wildlife balance these trade-offs in anthropogenic environments is crucial to develop effective strategies to reduce risks of negative interactions, including bi-directional aggression and disease transmission. For the first time, we use a landscape of fear framework with Bayesian spatiotemporal modelling to investigate anthropogenic risk-mitigation and optimal foraging trade-offs in Critically Endangered western chimpanzees (*Pan troglodytes verus*). Using 12 months of camera trap data (21 camera traps, 6722 camera trap days) and phenology on wild and cultivated plant species collected at Caiquene–Cadique, Cantanhez National Park (Guinea-Bissau), we show that humans and chimpanzees broadly overlapped in their use of forest and anthropogenic parts of the habitat including villages and cultivated areas. The spatiotemporal model showed that chimpanzee use of space was predicted by the availability of naturalised oil-palm fruit. Chimpanzees used areas away from villages and agriculture more intensively, but optimised their foraging strategies by increasing their use of village areas with cultivated fruits when wild fruits were scarce. Our modelling approach generates fine-resolution space–time output maps, which can be scaled-up to identify human-wildlife interaction hotspots at the landscape level, informing coexistence strategy.

## Introduction

The majority of the world's terrestrial biodiversity is impacted by humans^1,2^. Anthropogenic activities, including hunting and forest conversion to agriculture, pasture land and urban areas are causing significant wildlife population declines globally^3^. Hunting is a direct driver of biodiversity loss, particularly of large mammals, but also has indirect impacts on ecosystem functioning by fuelling trophic cascades^1^. Deforestation and habitat conversion lead to biodiversity loss indirectly through depletion of suitable habitat, food sources, loss of landscape connectivity and disruption of interspecific interactions and ecosystem cycles^4^. A recent study estimated that land use change and hunting caused an average 53% reduction to large mammal distribution in tropical regions between 1992 and 2015^5^. Human-induced land use change is now prevalent across the globe and agroforest mosaics represent one of the most extensive anthropogenic biomes in Africa, Europe and Asia^6^. Agroforests may represent permanent habitats to wildlife species, or temporarily accessed for supplementary food sources and/or to move between intact habitats ^7^. Low-intensity land uses mixed with remnant forests such as agroforest systems, are therefore increasingly recognised as integral to biodiversity conservation^7–11^.

Shared agroforest landscapes present a complex combination of costs and opportunities to wildlife. As a response to a reduction in wild space and resources, ecologically flexible species may access nutrient-rich human resources such as cultivated foods and/or livestock^12^. On the flipside, coexisting with humans increases risks of mortality and physical injuries. Direct risks include targeted hunting and retaliatory killings, but also accidental collisions with road vehicles^13^. Other risks may include snares and traps used for hunting or crop protection, reduced availability of wild food sources as well as increased stress and exposure to pathogens^14–17^. The presence of people and associated threats may restrict animal movements, and spatial constraints may result in loss of feeding opportunities at the small-scale and the ability to disperse at the larger scale, ultimately leading to population isolation, loss of gene flow and extirpation^4,18^. Understanding the strategies that wildlife use to balance costs and opportunities in shared landscapes, and how dynamics change over time, can facilitate long-term human-wildlife coexistence capacity.

The landscape of fear is an established concept in predator–prey interaction studies^19–21^. It posits that animals learn about spatiotemporal variations in risk through predation escapes, and the learnt fear shapes their decisions over where and when to feed, travel and rest^22^. There is a growing literature that incorporates humans as agents for shaping animals' landscapes of fear^23–26^. Most research to date has focused on the effects of a human-induced landscape of fear on predator–prey interactions, as well as ungulate species targeted by human hunters. Studies have found that the landscape of fear of prey, including elk/red deer (*Cervus elaphus*) and wild boar (*Sus scrofa*), is influenced more by human activities than the activity of wild predators^23,24^. Additionally, human activity and noise affect the spatiotemporal activity of many predators^26–29^. Much of the nonhuman primate research has focused on measuring human impacts on species distribution, abundance and habitat use^30–33^, and has examined responses to perceived anthropogenic risk^25,34^. Although the landscape of fear framework has been applied to predator-primate studies^19,20^, it has rarely been used within the context of human-primate interactions^25^. Considering over 60% of primates are threatened with extinction mainly due to agricultural expansion^35^, and that primate ecological adaptations often impact people’s livelihoods, a flexible framework for capturing dynamic human-primate interactions is needed.

In West Africa, nearly all habitat occupied by the Critically Endangered western chimpanzee (*Pan troglodytes verus*) is influenced by humans^36^. As a large-bodied, innovative and socio-ecologically flexible species, chimpanzees are able to adapt to complex and dynamic environments^37^ including anthropogenic landscapes^38,39^. Chimpanzees may generally be less risk-sensitive than other great apes^40^ with a higher propensity to undertake ‘risky' activities such as crop feeding. In anthropogenic landscapes, chimpanzees are known to incorporate a wide variety of cultivated food sources into their diet^41^, and are able to maintain an energy-rich largely frugivorous diet during periods of wild fruit scarcity by increasing the intake of cultivated fruits^42–44^. However, crop feeding behaviours are sometimes met with aggressive confrontations by farmers, including retaliatory killings, rock throwing and/or shouting and chasing. Antagonistic behaviour by humans towards chimpanzees is likely to induce a landscape of fear, which can be manifested in observable responses, including spatiotemporal adjustments. Where hunting occurs or has occurred in the past, chimpanzees have been shown to spatially avoid humans^45,46^. Even where chimpanzee-targeted hunting is low or absent, chimpanzees may exhibit fine-scale spatiotemporal adjustments, including feeding on cultivated foods at night^47^ and preferring to spend more time in the forest^30^.

A human-influenced landscape of fear will be impacted by human behaviour and tolerance towards wildlife. In areas with heavy hunting or persecution, animals are expected to maximize avoidance and/or become very cryptic. In some places, primates including chimpanzees are culturally important and afforded high levels of tolerance^48,49^. Some primates may even be fed by humans^50^. Positive behavioural feedbacks coupled with feeding opportunities result in closer interactions and loss of fear of humans, sometimes even leading to aggressive behaviour by primates towards people^50,51^. In agroforest contexts, the economic value of a cultivated food and the behaviour and extent of the damage by crop foragers will impact human tolerance and behaviour towards chimpanzees^52,53^. Aggressive or lethal behaviour by humans towards crop foraging wildlife may be used only temporarily and depending on the cultivated food involved. Complex human-wildlife dynamics are therefore impacted by long-term feedback loops, can undergo discrete changes (e.g. due to sudden human socio-political, ecological or economic shifts), and may seasonally differ (e.g. due to seasonal variations in wild and cultivated food availability or human activity)^54,55^. Many human-wildlife scenarios will involve wildlife balancing between risk-avoidance and feeding opportunities within measurable spatiotemporal scales^56^.

Humans and chimpanzees have been shown to use the same resources within agricultural zones, villages^57^, abandoned settlements^58^ and the forest^59^. In the agroforest landscape of Caiquene–Cadique, Guinea-Bissau, chimpanzees and humans overlap in the selection of 27 wild fruit species^59^. Therefore, although chimpanzees may spend more time within the forests^30^ compared to areas where human activities are more frequent such as villages and agricultural areas, human-chimpanzee interactions can occur across different habitat types and anthropogenic gradients. Human-induced risk sensitivity in chimpanzees within a particular location or habitat type will likely depend on the type and spatiotemporal intensity of human activity and behaviour at the particular location or habitat. In addition, feeding opportunities in cultivated areas will likely affect chimpanzee risk-taking behaviour, particularly during periods when wild foods are scarce. Chimpanzee spatiotemporal response to risks will therefore be conditional on spatiotemporal variations in human activity and availability of resources^42^. Spatial patterns will not remain constant over time because an optimal strategy must be flexible to variable conditions^43,60^. Finding efficient ways to monitor fine-scale human-chimpanzee spatiotemporal interactions and ecological trade-offs in anthropogenic habitats is crucial to develop evidence-based strategies that promote long-term coexistence^36^.

Here, using a landscape of fear framework we employ Bayesian spatiotemporal modelling using the Integrated Nested Laplace Approximation (INLA) algorithm^84,85^ to examine how chimpanzees balance risk from humans and access to food within an agroforest landscape. Our approach allows us to:

1. Simultaneously identify the effects of risk and resource availability that shape spatiotemporal variations in animal range use;
2. Identify constraints that species may impose on one another and coexistence opportunities in human-influenced landscapes;
3. Predict when and where close human-wildlife interactions and potential negative interactions over resources are more likely to occur;
4. Generate a model output in the form of fine resolution space–time maps to facilitate targeted conservation interventions in shared landscapes.

**Figure 1:**
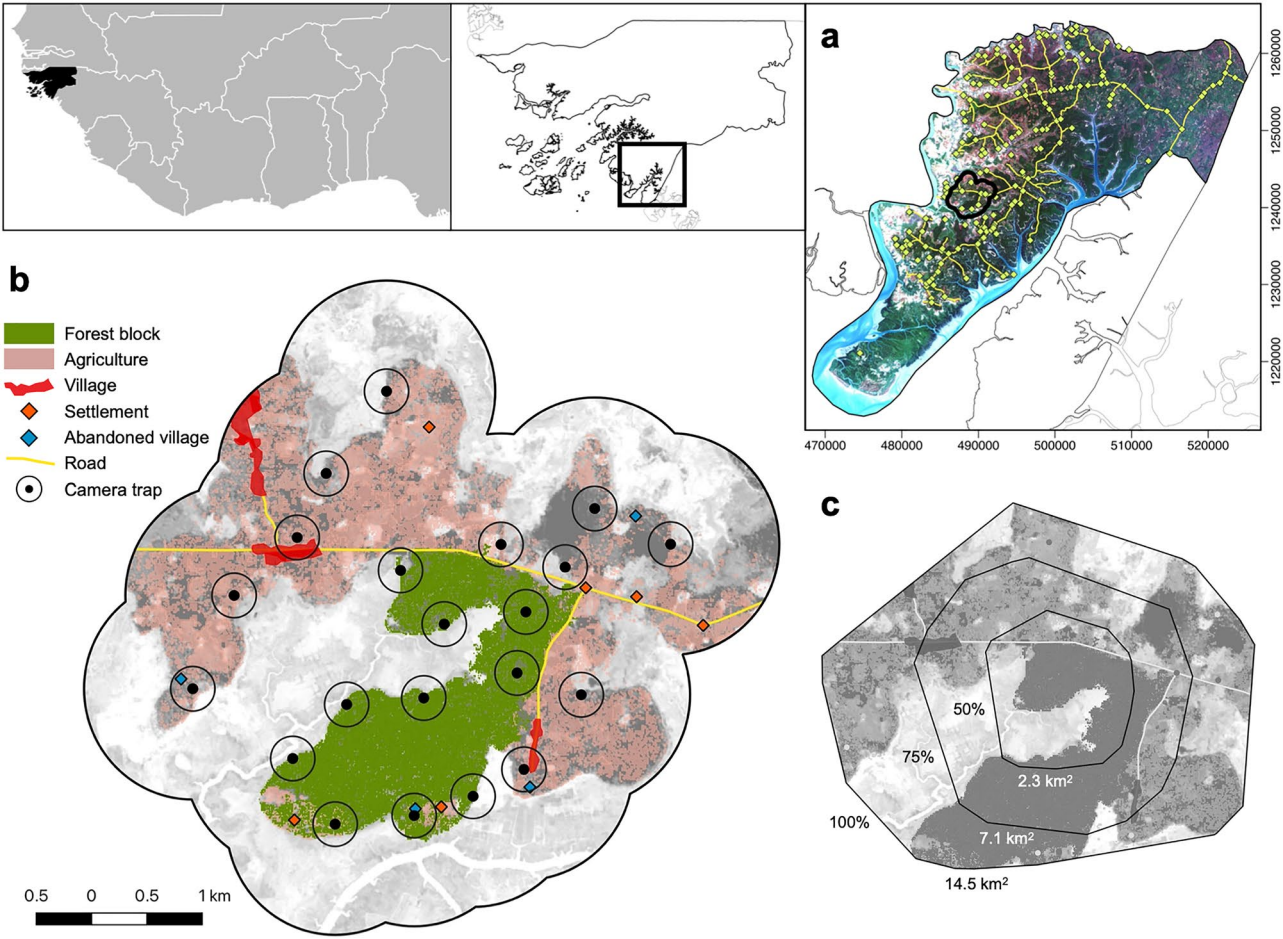
Map and location of the study area in Caiquene–Cadique, Cantanhez National Park, Guinea-Bissau, West Africa. (**a**) Cantanhez National Park includes a road network and approximately 200 villages and settlements. (**b**) Map of Caiquene–Cadique study area showing the 21 camera trap sampling sites and respective 200 m buffers where availability of chimpanzee foods was quantified. The forest block is shown in green and the heterogeneous matrix outside the forest block includes cashew orchards and shifting cultivation fields shown in pink, roads, three villages, five settlements and four abandoned villages. (**c**) Estimated home range of Caiquene–Cadique chimpanzees using 100% minimum convex polygon (MCP) analysis of 1380 direct and indirect observations of chimpanzees collected between 2013 and 2018. MCP analyses were ran using the R package ADEHABITATHR version 0.4.19^67^. The base layers in a-c panels consist of modified Copernicus Sentinel-2 data from 25 January 2017 (RGB colour and grey scale). Sentinel-2 imagery was downloaded from the Sentinel Hub, Sinergise Ltd (https://www.sentinel-hub.com/). All maps were created using QGIS version 3.10.5 (https://www.qgis.org).

We completed a 12-month camera trap and phenological monitoring survey within the home range of one focal chimpanzee community at Cantanhez National Park (NP), Guinea-Bissau (Fig. 1). Cantanhez NP covers 1057 km^2^ and includes approximately 24,000 human inhabitants^61^. Despite the relatively high human density (c. 23–26 people km^−2^ in Cantanhez NP), sufficient landscape connectivity and low levels of direct persecution towards chimpanzees allows them to inhabit human-impacted areas^62^. Similarly to several sites across West Africa, chimpanzees in Cantanhez NP are not hunted for consumption due to cultural beliefs and religious taboos^49^ but are sometimes killed in retaliation for foraging on cultivated foods, killed and captured to be sold as pets, and/or suffer injuries from snares set up by farmers to catch smaller animals. The Caiquene–Cadique study area coincided with the home range of one of the approximately twelve chimpanzee communities present in Cantanhez NP^63^. The Caiquene–Cadique chimpanzees include at least 48 individuals ranging across approximately 14.5 km^2^^59,64,65^. Chimpanzees in Caiquene–Cadique are known to feed on cashew pseudo fruit, available across much of the agricultural matrix, as well as mango and baobab present in villages and abandoned villages, and orange, lime and papaya grown within villages^59,63,64,66^.

We tested the following hypotheses: (1) chimpanzees and humans overlap in space use but chimpanzees maximize spatial partitioning preferring the forest block to the agroforest matrix, indicating a chimpanzee home range use strategy largely driven by spatial risk-avoidance; (2) chimpanzee spatiotemporal home range use is driven by both risk avoidance and resource availability, consistent with a landscape of fear that balances between feeding and risk-avoidance strategies; (3) chimpanzees increase the use of high-risk areas when cultivated foods are available and wild foods are scarce, providing evidence of temporal trade-off in favour of optimal foraging.

To test for evidence of spatial partitioning between chimpanzees and humans (hypothesis 1), we used relative detection frequencies (RDF), measured as the number of independent events scaled to the number of camera trap days multiplied by 100. We predicted a negative correlation between human and chimpanzee RDFs across camera trap sampling sites and higher chimpanzee RDFs within the forest block compared to the agroforest matrix. To test for hypotheses 2 and 3, we fitted spatiotemporal models using the chimpanzee intensity of space use (detection frequencies) as the response. To support a balanced risk-avoidance/optimal foraging strategy (hypothesis 2), we expected the intensity of space use by chimpanzees to be negatively influenced by human factors (villages, agriculture, roads and/or human detections) and positively predicted by the availability of resources (wild and/or cultivated). To test for dynamic risk-avoidance/optimal foraging trade-offs and account for risk differences associated with resources (hypothesis 3), we grouped cultivated fruits into three risk gradients with respect to their availability and locations across the landscape. From lower- to higher-risk, these were (i) cashew fruit, the most abundant cultivated food, available across much of the matrix including far and close to villages, (ii) mango and baobab fruits combined, available in both villages and abandoned villages and finally (iii) orange, lime and papaya fruits, available only in villages and at low abundance. We identified cultivated foods available during wild food scarcity periods by checking the temporal patterns of availability of cultivated resources in relation to wild foods. We predicted a positive effect of the cultivated foods available during periods of wild fruit scarcity on chimpanzee intensity of space use.

## Results

### Human-chimpanzee overlap

**Figure 2:**
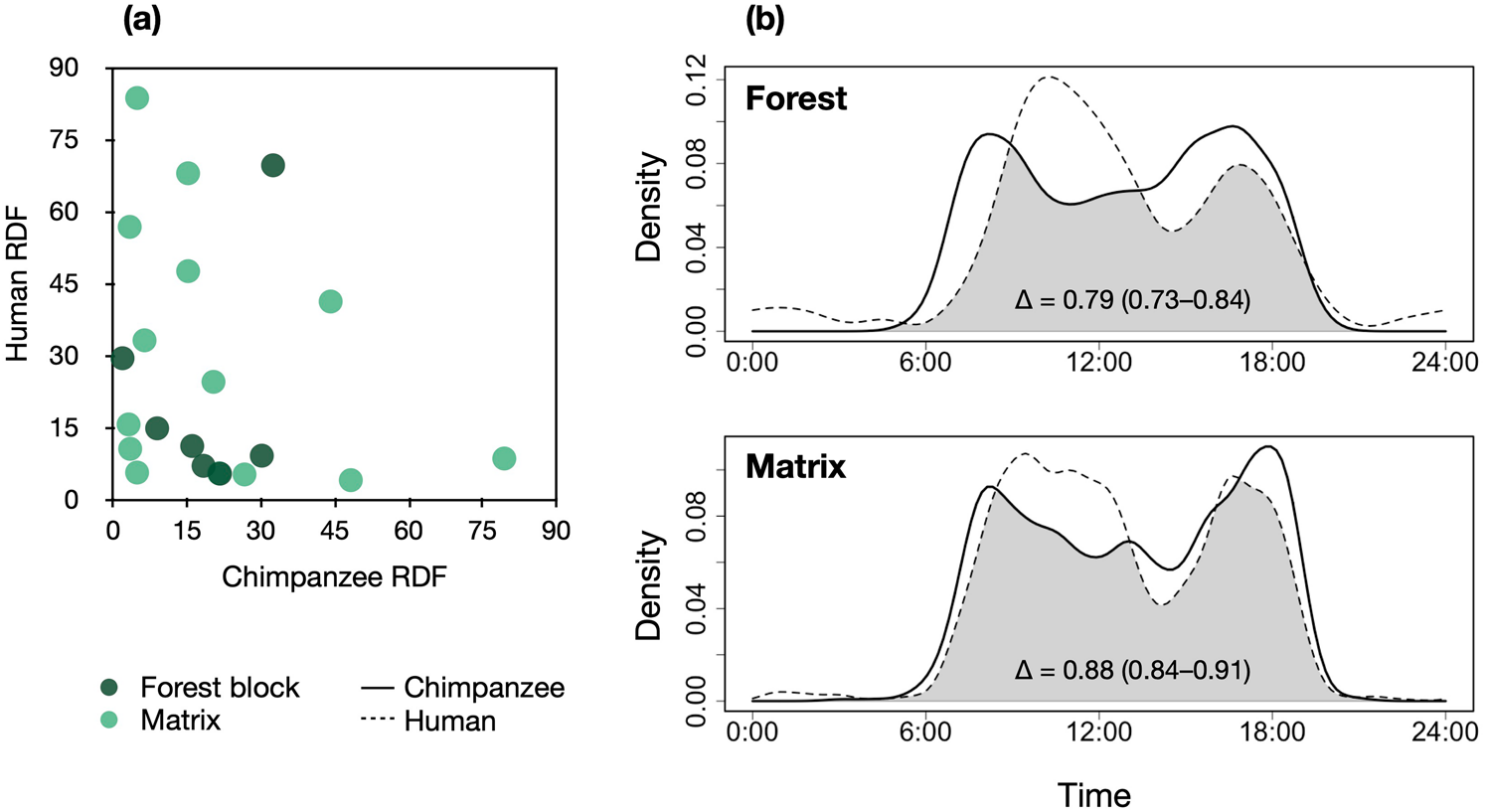
Comparison of relative detection frequency (RDF) (**a**) and temporal activity patterns (**b**) between chimpanzees and humans during a 12-month period across 21 camera trap sampling sites in Caiquene–Cadique. ∆ is the coefficient of overlap^68,69^. Values in brackets represent 95% confidence intervals.

Chimpanzees and humans showed variation in intensity of space use across locations both within the main forest block and the heterogeneous agroforest matrix (Fig. 2a). We found no evidence of spatial partitioning between chimpanzees and humans. There was no correlation between the RDFs of chimpanzees and humans during the 12-month sampling period and across the sampling sites (*r*_*s*_ = − 0.359, N = 21, *P* = 0.1098). We identified an upper limit of co-occurrence; there were no sampling sites where both chimpanzee and humans had > 45 RDF values. We found no correlation between overall chimpanzee and human RDFs at sites within the forest blocs (*r*_*s*_ = − 0.1428, *P* = 0.7825) nor across the matrix (*r*_*s*_ = − 0.4066, *P* = 0.1505). Overall RDFs across the 12-month study did not differ between the forest and matrix for chimpanzees (Wilcoxon-test: *W* = 54, P = 0.7433) nor humans (*W* = 44, P = 0.7433). Temporal overlap between chimpanzees and humans was higher across the heterogeneous matrix compared to within the forest block (Fig. 2b). Periods of activity for chimpanzees and humans differed significantly within the forest block (Wald-test for activity levels: *w* = 5.195, *P* = 0.023), and not across the matrix (*w* = 0.156, *P* = 0.693). In general, humans were detected in the forest mainly during late-morning hours and compared to chimpanzees, people were more active in the forest at night.

### Chimpanzee food availability

**Figure 3:**
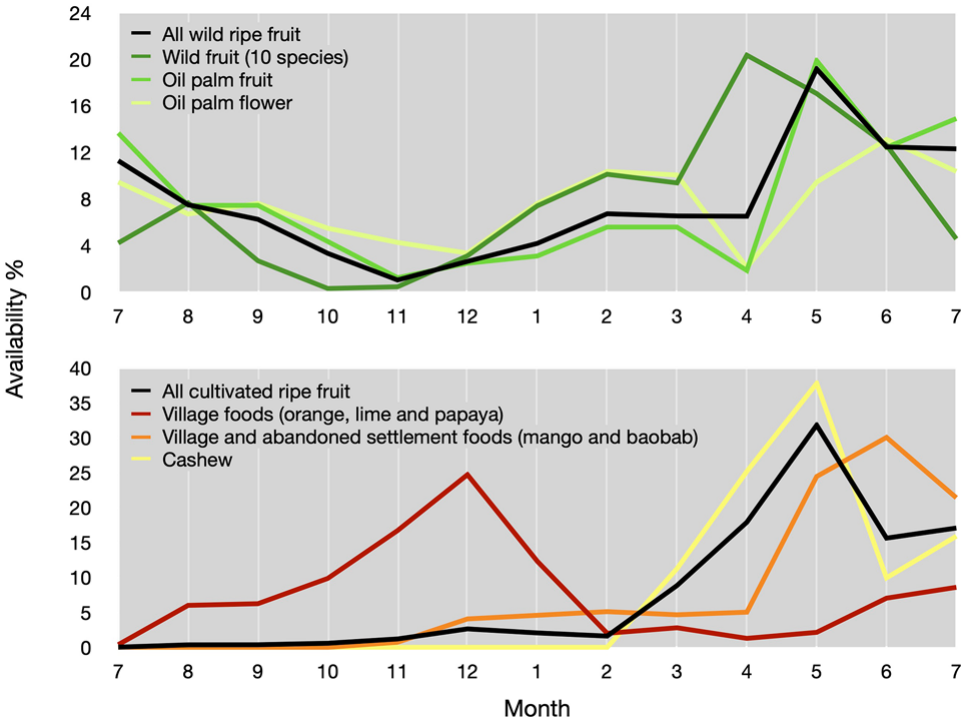
Monthly variation in availability of chimpanzee key food sources in 2017–2018 in Caiquene–Cadique, Cantanhez National Park. Frequency of availability is the mean availability index of resource or group of resources *k* scaled by the total availability during the study period (12 months). Village foods include the combined availability of orange, lime and papaya ripe fruit; village and abandoned settlement foods include the combined availability of mango and baobab ripe fruit; wild fruit include the combined availability of ripe fruit of ten important chimpanzee food species (Supplementary Figs. S2–S3).

Across the Caiquene–Cadique chimpanzee home range we identified a total of 98 wild and 10 cultivated plant species with diameter at breast height (DBH) ≥ 10 cm (total sampled area 90,600 m^2^, N of 200 m^2^ plots = 453). On average, plant species diversity was higher within the forest block (Shannon diversity index ± SD: 0.74 ± 0.61) compared to across the heterogeneous matrix (0.47 ± 0.58). Cashew was the most abundant plant species measured (overall density of 22 stems/ha), followed by wild oil palm (*Elaeis guineensis*, 14.6 stems/ha). Excluding oil palm, the density of most important wild plant species consumed by chimpanzees was higher within the forest (38.7 stems/ha, N species = 8) compared to the matrix (13.1 stems/ha, N species = 9), whereas oil palm density was similar inside and outside the forest block (13.8 and 11.9 stems/ha, respectively). The temporal availability of chimpanzee foods fluctuated across the months (Fig. 3). Wild ripe fruit availability was highest from April through June, also coinciding with peaks in cashew and mango ripe fruit. The period of wild fruit scarcity (October–January) coincided with the peak in ripe fruit cultivated within villages (orange, lime and papaya). The inverse relationship between wild fruit and village (high-risk) foods allows us to use availability of village foods as a proxy for risk trade-offs during times of wild food scarcity.

### Spatiotemporal patterns of chimpanzee home range use

Of the 20 models considered (Supplementary Table S5), the model with the lowest Deviance Information Criterion (DIC) included eight covariates (Table 1). Chimpanzee spatiotemporal home range use was driven by both risk of exposure to humans and opportunity to access food resources. At the spatial dimension, proximity to villages and agriculture negatively influenced intensity of space use by chimpanzees and in contrast, areas in proximity to roads positively predicted chimpanzee space use. At the spatiotemporal dimension, intensity of space use by chimpanzees was unaffected by that of humans. Regarding wild chimpanzee foods, the availability of ripe oil palm fruit positively predicted chimpanzee space use. As for the cultivated chimpanzee foods, the availability of ripe mango and baobab fruit combined showed a negative effect on chimpanzee space use, indicating that chimpanzees use areas characterized by low availability of mango and baobab (abandoned villages) more frequently than areas with high availability of these fruits (villages). In contrast, the availability of village-only fruits (orange, lime and papaya) showed a positive effect on chimpanzee intensity of space use.

**Table 1:** Posterior estimates of the chimpanzee spatiotemporal model. Values show the posterior mean of the intercept and covariate coefficients *β* for the final model, including standard deviation, credible interval quantiles and mode. Bold indicates covariates where credible interval does not cross the zero value. (Final model DIC = − 737.2343, compared to no-covariate model DIC = − 683.9679, ΔDIC = 53.27. See Supplementary Table S5 or the full list.).

Covariate	Mean	± SD	2.5%	50%	97.5%	Mode
Intercept	0.158	0.023	0.112	0.158	0.203	0.158
**dist_village**	**0.087**	**0.025**	**0.038**	**0.087**	**0.136**	**0.086**
**dist_road**	− **0.103**	**0.025**	− **0.153**	− **0.103**	− **0.054**	− **0.103**
dist_agriculture	0.034	0.021	− 0.007	0.034	0.074	0.034
human_det	− 0.001	0.015	− 0.029	− 0.001	0.028	− 0.001
**palm_fruit**	**0.022**	**0.011**	**0.001**	**0.022**	**0.043**	**0.022**
ten_wild_fruits	0.005	0.011	− 0.016	0.005	0.027	0.005
mango_baobab	− 0.015	0.013	− 0.041	− 0.015	0.012	− 0.015
orange_lime_papaya	0.012	0.013	− 0.014	0.012	0.039	0.012

dist_village = linear distance to the nearest village; dist_road = linear distance to the nearest road; dist_agriculture = linear distance to the nearest agricultural area (including cashew orchards, shifting cultivation fields and mangrove rice cultivations); human_det = human detection frequencies, or human intensity of space use including missed observations as predicted by the human spatiotemporal model (Supplementary Table S3); palm_fruit = availability of ripe oil palm fruit; ten_wild_fruit = availability of ten wild fruits combined; mango_baobab = availability of ripe mango and baobab fruit combined; orange_lime_papaya = availability of orange, lime and papaya combined (village-only foods).

**Figure 4:**
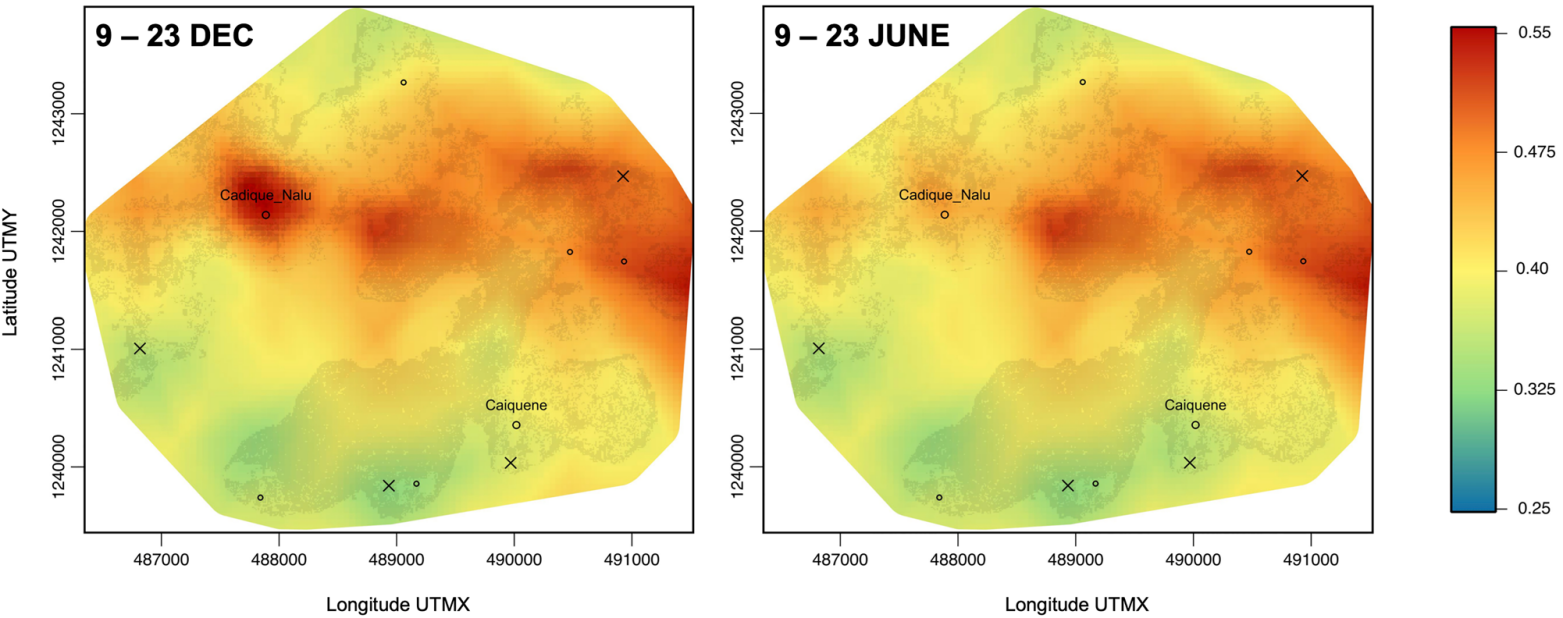
Predicted chimpanzee intensity of space use across their home range in Caiquene–Cadique in two time periods. December 2017 corresponded to the peak in availability of village fruits (orange, lime and papaya), June 2017 coincided with the peak in mango ripe fruit and wild fruits (Fig. 3). Circles indicate the location of villages (Cadique Nalu and Caiquene) and small settlements (unlabelled smaller circles); crosses represent abandoned villages. Prediction raster layers were created using the R package RASTER version 3.4-5^70^. Figures were created using QGIS (https://www.qgis.org). See Movie S1 for predictions of chimpanzee intensity of space use across the 12-month study period.

Our model output showed higher intensity of chimpanzee space use across the heterogeneous matrix compared to much of the southern part of the forest block (Fig. 4). According to the mapped predictions, at the peak of the orange fruiting season chimpanzees intensified their use of space in and around Cadique Nalu village, where we recorded the highest availability of village-only fruit (orange, lime and papaya). In June, when oranges were unavailable and the availability of mangoes and wild food was high, the model predicted lower use of this village. Movie S1 shows chimpanzee space use predictions over the 12-month study period.

## Discussion

We employed Gaussian Markov Random Field (GMRF) Bayesian modelling with INLA^85^ to measure and map spatiotemporal interactions between humans and wild chimpanzees. To our knowledge, this was the first study to make use of this modelling approach for fine-scale, camera trap-based georeferenced data, and the first non-experimental study on a nonhuman primate to use a spatially explicit human-induced landscape of fear framework. Considering the ever-increasing number of ecological studies employing camera traps (1525 entries when searching for "camera trap" in Web of Science, 27 January 2021), our approach is relevant to any human-impacted forest system across the globe.

As demonstrated for some large-bodied and potentially dangerous carnivores that share the same space and resources with humans^27,71^, we found no evidence for a strong spatial separation between chimpanzees and humans, hence reject hypothesis 1. In Caiquene–Cadique, humans and chimpanzees share at least nine cultivated foods and 27 wild fruit species^59,64^. Some wild foods including oil palm, *Ficus* spp., *Dialium guineense* and *Saba senegalensis* are intensively used by both humans and chimpanzees^59^. Similarly, in Hawf, Yemen, human presence predicted the occupancy of some taxa including hyenas (*Hyaena hyaena*) and wolves (*Canis lupus*), likely due to sharing the same water points across the landscape and the presence of livestock and human leftovers constituting attractive food sources^71^. The fine-scale co-occurrence between chimpanzees and humans in Caiquene–Cadique across multiple habitats is most likely associated with the high overlap in the use of footpaths to access food resources, including wild fruits available in the forest block and both wild and cultivated resources available across the heterogeneous matrix^59,63,64^.

Despite chimpanzees not avoiding people at the sampling site-level, the spatiotemporal model showed a chimpanzee preference for areas away from villages and to a lesser extent agricultural areas, two important risk-related spatial landmarks within the chimpanzees' home range. Villages are characterized by the most intense levels of human activity and although they were accessed by the chimpanzees, villages represented the highest risk-peaks within their home range. Cashew orchards have similar canopy cover to a woodland habitat and are used by chimpanzees to travel across the matrix, feed on cashew fruit when available^66^ and wild foods that may be present within the orchards. Non-arboreal shifting cultivation fields mainly used for peanut, bean, cassava and rice farming are also present across the matrix and chimpanzees sometimes use them to feed on sugar cane, pigeon peas and hibiscus^64^. Our model reflects these previous findings but also suggests some spatial constraints in chimpanzees’ use of agricultural fields. Local people in Cantanhez have described how chimpanzees stop using or divert their path around newly established cultivated fields^63^.

**Figure 5:**
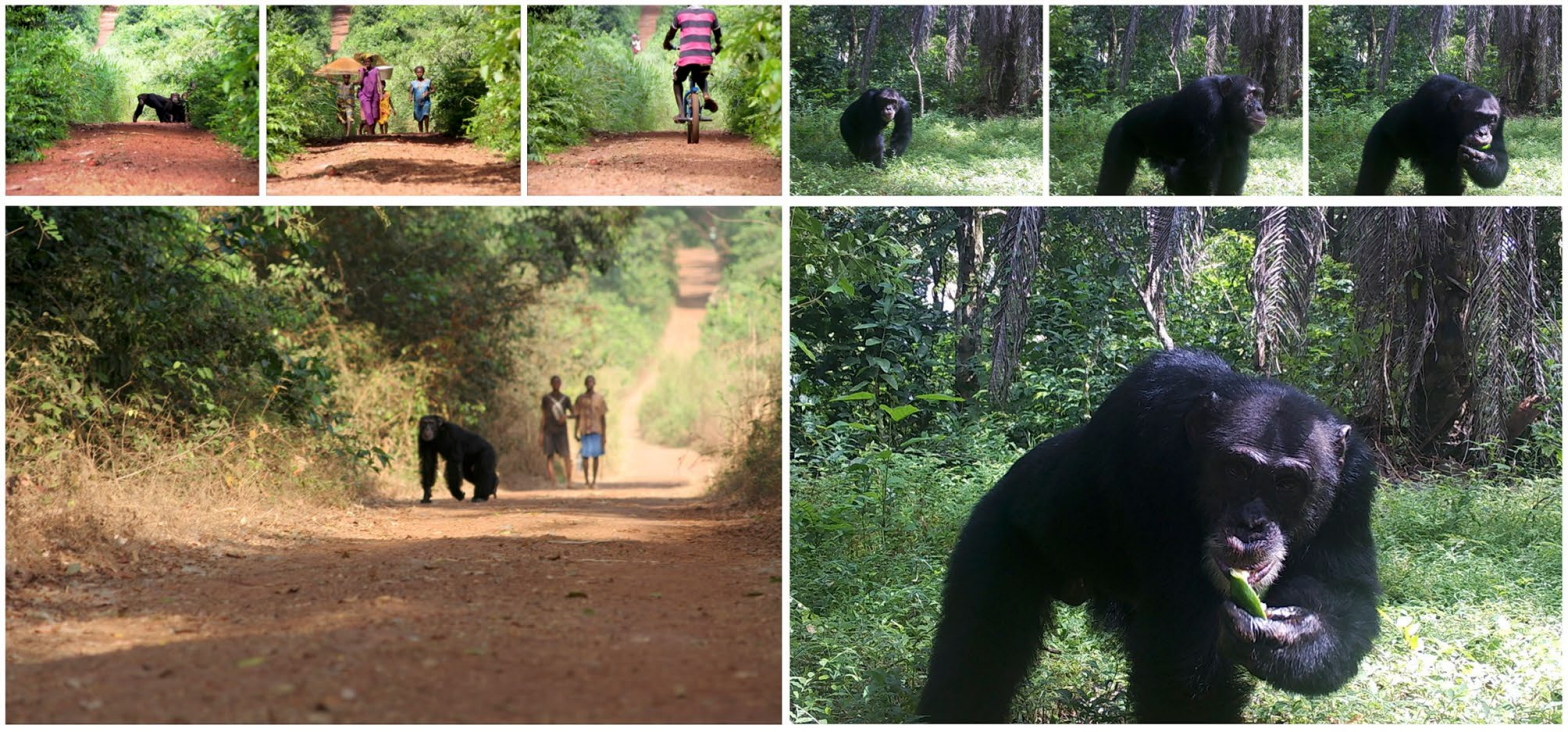
Chimpanzees in Caiquene–Cadique often use the roads located at the centre of their home range and frequently feed on cultivated foods. The camera trap images (right) show a chimpanzee entering Caiquene village to feed on orange in October 2017.

Strikingly, mapped predictions showed highest levels of chimpanzee space use across the central and eastern section of their home range, most of which is associated with heterogeneous forest-savannah-agriculture mosaics including roads. Our results indicate that at present, the small roads in Caiquene–Cadique do not pose any spatial constraints to chimpanzees. These two gravel roads are located within the chimpanzees’ core range. A long section (c. 1.8 km) of these roads borders the forest block. At this site, chimpanzees are frequently seen by local people crossing these roads (Fig. 5) and multiple chimpanzee crossing points can be discerned where shrub/forest edges are present (unpubl. data). Our results contrast with data from Moyamba district, Sierra Leone, where chimpanzees avoid roads; however, roads there are mostly surrounded by shifting cultivation fields^33^.

During our study wild oil palms were the second-most abundant plant species in Caiquene–Cadique present across the forest block and matrix. The availability score of oil palm fruit contributed disproportionally to the combined availability of all eleven wild fruits (Supplementary Fig. S3). Our findings reiterate the ecological importance of oil palm for this chimpanzee community^64,72^, supporting similar findings elsewhere^42,73,74^. We also demonstrate that chimpanzees are able to exploit and benefit from the presence of wild foods across the heterogeneous agroforest matrix.

We have shown that in Caiquene–Cadique chimpanzee spatiotemporal variation in home range use is shaped by both risk and food availability, supporting a moderate landscape of fear model (hypothesis 2). We also demonstrate that spatial sensitivity to risk changes depending on food availability, highlighting the importance of including a temporal dimension. Village foods peaked when the rest of the landscape had little to none of the most important chimpanzee foods. As displayed by the prediction maps, the positive relationship between village foods and chimpanzee intensity of space use indicates that when wild foods are scarce, chimpanzees will temporally override potential risks in favour of optimal feeding, supporting hypothesis 3. Our study strongly suggests that chimpanzees access high-risk orange, lime and papaya fruits in response to nutritional necessity rather than preference alone. When entering the “risky” village, the Caiquene–Cadique chimpanzees are likely to use behavioural strategies to mitigate risks, including increased vigilance, food transport and different social grouping patterns such as forming larger party sizes, increasing cohesiveness and/or the number of males within the party^38,57,63,75,76^.

Human-induced risk-free space is increasingly rare for the majority of wildlife including primates^7,35^. The capacity to ensure long-term human-wildlife coexistence will only be achieved with evidence-based strategies that integrate the costs and benefits of sympatry and monitor dynamics over space and time. The landscape of fear framework integrates ecological constraints and opportunities when measuring animal ecological and behavioural responses. We show that a spatiotemporal approach can produce fine-scale maps relevant to inform targeted and inclusive conservation strategies. Our model output from Caiquene–Cadique clearly highlights potential mechanisms of competitive interactions at the village level. These data can be extrapolated at the larger scale, provided there are similar human and ecological conditions. Across Cantanhez NP, working with local people to address negative human-chimpanzee interactions in villages will likely have a larger impact for chimpanzee conservation rather than focusing on protecting forest blocks alone. Fine-scale human-wildlife interaction maps can be used in land use planning, and inform discussions about cultivated food (re)location initiatives with farmers, as well as forest restoration programs and planting of wildlife food sources away from villages. Our model is relevant to other sites where people and wildlife coexist by providing a framework for understanding and mitigating negative interactions through measuring real-time spatiotemporal interactions. Our approach will also be useful to monitor and evaluate conservation interventions such as those related to the spatial management of wild and agricultural plant resources. Including humans in ecological frameworks is crucial to understand how different species, including threatened great apes, are able to persist in changing environments. A spatiotemporal landscape of fear approach provides the necessary tools to understand and more effectively manage human-wildlife coexistence at different spatial scales, including the management of resources important to both.

## Materials and methods

### Study area

Guinea-Bissau (36,125 km^2^), West Africa, lies within the Guinean forest-savannah mosaics, a biodiverse ecoregion buffering the Guinean moist forests in the south and the West Sudanian savannah in the north^77^. The climate in Guinea-Bissau is characterized by a rainy season from mid-May to the end of October and a long dry season from November to mid-May. Cantanhez NP (N11° 14.287′ W15° 02.281′) comprises the Cubucaré peninsula in the Tombali Region bordering Guinea-Conakry. The landscape in Cantanhez NP consists of a mosaic of coastal sub-humid forest patches, mangroves, savannah grassland, woodland and agriculture including mostly cashew orchards, shifting cultivation fields and mangrove swamp rice fields. Approximately 24,000 people across 200 villages and settlements are present inside the park (Fig. 1a). At the time of this study, Cantanhez included a network of small gravel roads (up to approximately 6 m wide) and most of them were in poor condition. The majority of traffic consisted of people traveling on foot, bicycle and motorbike. Residents in Cantanhez NP include mainly Balanta, Nalu and Fula people but also Tanda, Sussu, Mandinka, Pepel and Djakanka among others. Most people in Cantanhez are Muslim except from people of Balanta and Pepel ethnicity who mainly practice animism and/or Christianity.

### The Caiquene–Cadique chimpanzees

The landscape in Caiquene–Cadique comprises a main protected forest block covering 3 km^2^ and a matrix characterized by a mosaic of forest remnants (totalling approx. 2.3 km^2^), palm groves (1.6 km^2^), mangroves (0.9 km^2^), grassland (3 km^2^) and anthropogenic land uses including shifting cultivation fields, cashew and orange orchards, villages, settlements and roads (4 km^2^, Fig. 2b)^63^. The three villages present, namely Caiquene, Cadique Nalu and Cadique Iala, included 141, 322 and 718 inhabitants respectively in 2009^61^, with an additional five smaller settlements (2–34 inhabitants in each)^59^. Chimpanzees at Cantanhez NP including Caiquene–Cadique are not habituated to researchers precluding behavioural follows. The Caiquene–Cadique chimpanzees have been studied by the Cantanhez Chimpanzee Project since 2013. Based on direct observations of chimpanzees during road crossing in 2013–2018 and on camera trap individual identification since 2015, the Caiquene–Cadique chimpanzee community includes 48 or more individuals^59,64^. Their home range is estimated to cover approximately 14.5 km^2^ and includes Caiquene, Cadique Nalu and the five settlements using 100% MCP analysis of 1380 direct and indirect observations (camera traps, nests, feeding traces, faeces) from 2013 to 2018 (Fig. 1c). Based on visual analysis of faecal samples the Caiquene–Cadique chimpanzees feed from at least 66 wild plant species, from which mainly fruit (64.3%) and leaves (20.2%) were recorded but also pith (8.3%), bark (3.6%), flower (2.4%) and sap (1.2%)^64^. Besides consuming naturalised wild oil palm fruit, flower and pith^64^, oil palms are the most common tree selected for nests by chimpanzees in this community^72^. Caiquene–Cadique chimpanzees also feed on honey relatively frequently (8% of samples) and fruit, leaves and/or pith from at least nine different cultivated foods^64^. Chimpanzees at Cantanhez are not hunted for food because of their perceived similarity to humans in general^78^, and because of taboos against the consumption of primates in Islam, including chimpanzees. In addition, chimpanzees are important in Nalu and Balanta culture^49^. Chimpanzees are sometimes killed in retaliation to crop feeding particularly when involving orange fruit. The Caiquene–Cadique chimpanzees are one of at least three chimpanzee communities in Cantanhez NP affected by *Mycobacterium leprae*, and include two adult females showing advanced-stage leprosy disease^65^. Despite the high human-chimpanzee spatiotemporal overlap at Cantanhez, current evidence does not point to a direct human-to-chimpanzee transmission as the origin of leprosy infection; the rarity of the genotype of *M. leprae* suggests a possible environmental or animal source yet to be identified^65^.

### Ethics declaration

This research involving wild unhabituated chimpanzees was non-invasive and complied with the ethics guidelines detailed by the Association for the Study of Animal Behaviour (UK) and to the legal requirements of Guinea-Bissau in which the research was conducted. Formal permission to conduct research in Cantanhez NP was obtained from the Instituto da Biodiversidade e das Areas Protegidas (IBAP) in Bissau. Research permission was also obtained from local authorities in Cadique Nalu and Caiquene villages. The collection and management of camera trap data including people was approved by the University Research Ethics Committee at Oxford Brookes University (permission granted on 28 June 2016, UREC Registration No: 161018).

Obtaining permission from local residents within study areas and clear communication of research objectives and data anonymity are essential to ensure that research within human-wildlife ecosystems is carried out ethically^79^. To obtain permissions from local residents, prior to camera trap deployment we met with the regional chief (resident of Cadique Nalu), the Caiquene village committee, as well as individual farmers and residents of settlements to seek permission to set up camera traps within their area of jurisdiction. We first described the objectives of our research and how camera traps worked, including information about the camera trap field of view and triggering system, so that local residents understood what the camera traps could do. We stated that a camera trap would record images when an animal or person would pass in front of it, so that residents were aware of the likely possibility of being photographed by camera traps, particularly when deployed on forest footpaths and orchards. We informed residents that camera traps were used with the purpose of conducting research on animal movement across the landscape in relation to habitat type and human land use activities. We stated that all camera trap photographic information was to remain anonymous, images would be viewed solely by EB and data stored by EB securely. Once camera traps were deployed, we again visited the villages and settlements and informed residents about the locations of the camera traps. All camera traps in cashew and orange orchards were deployed with the permission and involvement from the orchard owners. For camera traps deployed within orchards or near shifting cultivation fields, farmers either accompanied us or were present during camera trap deployment. In two instances (Caiquene and Cadique Nalu), farmers offered to help us locate chimpanzee paths within their orange orchard. We informed residents that camera traps in orchards were likely to record people frequently, and that farmers were expected to go on with their business as usual to avoid disrupting farming activities. Again, we emphasised that camera trap data were to be kept entirely anonymous. Consent was obtained from all villages and settlements across the study area, including Cadique Nalu, Caiquene, Abdaia, Camada, Caras, Butchifula and Crozamento de Caiquene. All permission from local residents to deploy camera traps in their areas was obtained verbally.

### Data collection

We collected data on Caiquene–Cadique chimpanzees using 21 camera traps deployed across their home range (mean distance between camera traps: 652 m SD ± 115, range 520–918 m, Fig. 1). We set up camera traps pointing towards main chimpanzee paths in areas characterized by different human activities, including the main forest block and across the heterogeneous agroforest matrix: small forest remnants, cashew orchards, abandoned villages and in orange orchards in villages. Cameras were set up to record three consecutive photographs when triggered and remained active for 12 months between 9 July 2017 and 5 July 2018. The total effort was 6722 camera trap days (average camera trap days per sampling site: 320.10 days, SD ± 33.67, range 247–359 days).

We quantified availability of wild and cultivated chimpanzee foods via phenology trails and vegetation plots^38,43^. We monitored the most important wild (N = 11) and cultivated (N = 6) food tree species to this chimpanzee community based on previous feeding ecology data^59,64^ (Supplementary Table S1). The wild plant species included *Elaeis guineensis*, *Dialium guineense, Ceiba pentandra*, *Treculia africana*, *Parinari excelsa*, *Saba senegalensis*, *Spondias mombin*, *Detarium senegalense*, *Landolphia heudelotii*, *Ficus sur*, *Uvaria chamae*. Cultivated plants included cashew (*Anacardium occidentale*), baobab (*Adansonia digitata*), mango (*Mangifera indica*), orange (*Citrus sinensis*), lime (*Citrus aurantifolia*) and papaya (*Carica papaya*). Every two weeks we assigned a 0–4 score for new leaves, flowers, unripe and ripe fruit of 8 to 10 individuals for each tree species. To measure spatial variation in food availability, at each camera trap sampling site we gathered data from 25 10 × 20 m vegetation plots within a 200 m radius (4% of the sampled area). The length of the radius was chosen to maintain spatial independence between camera trap sampling sites and provide a representation of the availability of foods in the area surrounding each camera trap sampling site. Plot locations were pre-established randomly using the research tool ‘Random points inside polygon’ on QGIS, setting 50 m as a minimum distance between plots. Plots located in inaccessible or flooded areas (usually mangroves) or sacred forest were discarded. In total, we sampled 453 plots (90,600 m^2^).

### Data analysis

We calculated the monthly food availability index (*F*_*m*_) for the selected wild and cultivated chimpanzee food species using the formula:

$$\it {\text{F}}_{{\text{m}}} = \mathop \sum \limits_{{{\text{k}} = {1}}}^{{\text{n}}} {\text{F}}_{{\text{k}}} \times {\text{D}}_{{\text{k}}} \times {\text{S}}_{{\text{k}}}$$

where *F*_*k*_ is the mean score of ripe fruit of monitored individuals in species *k* in month *m*, *D*_*k*_ represents the overall density of adult trees in species *k* measured in plots, and *S*_*k*_ is the overall mean DBH of adult trees in species *k* measured in plots^43^. The reproductive minimum size of adult trees of different plant species was selected based on previous studies^80,81^ and from personal observations during fieldwork (Supplementary Table S1).

We calculated the relative detection frequency (RDF) as the number of independent events divided by the camera trap effort (number of days) and multiplied by 100 for each camera trap sampling site. Previous camera trap studies have used this measure and referred to it as the Relative Abundance Index (RAI; e.g.^82^). We chose to refer to it as ‘RDF’ instead of ‘RAI’ to reflect a measure of site selection rather than abundance. An event was considered independent from another event if the photograph or sequence of photographs were recorded 0.5 h before and after another photograph^83^. No attempt was made to identify individuals when calculating the number of independent events. The total number of independent events was 1392 for chimpanzees and 1734 for humans. Chimpanzee and human RDFs at each sampling site were not normally distributed (Shapiro–Wilk tests: w = 0.833, *P* = 0.0022; w = 0.826, *P* = 0.0017, respectively). We therefore used Spearman correlations to check for a negative relationship between chimpanzee and human RDFs across all sampling sites (N = 21), those within forest only (N = 7) and those across the matrix only (N = 14). We used Wilcoxon tests to test for significant differences of chimpanzee and human RDFs between the forest block and matrix. We extracted kernel density and compared activity patterns of chimpanzees and humans using the R package OVERLAP version 0.3.3^69^.

### Dataset for the spatiotemporal model

The 12-month camera trap dataset was divided into 24 sampling time-periods that matched the twice monthly phenological data collection. Due to calendar months varying in length, sampling occasions varied from 12 to 16 days (mean 15.1, SD ± 0.9). To select cut-off dates, we attempted to balance between consistency in occasion length and ensuring that the phenological sampling day fell approximately in the middle of the sampling occasion. For example, sampling period 1 started on the 9^th^ of July and ended on the 23^rd^ of July (15 days), corresponding with phenological data collected on the 15^th^ of July. When a camera was inactive for more than seven days during a sampling period, for example due to malfunction, we assigned "NA" to the sampling period. The total number of sampling periods per camera trap sampling site ranged from 17 to 24 (mean 21.5, SD ± 2.3).

We considered intensity of space use by chimpanzees as the response variable for the models, which consisted of the number of chimpanzee independent events at each camera trap sampling site during each sampling period, scaled to the number of camera trap active days. We considered the following covariates: (1) linear distance to the nearest village, (2) linear distance to the nearest road, (3) linear distance to the nearest agricultural area, (4) linear distance to the forest block, (5) human detection frequencies, (6) combined availability of eleven wild ripe fruit species, (7) availability of ripe cashew fruit, (8) combined availability of ripe mango and baobab fruit, (9) combined availability of ripe orange, lime and papaya fruit. In addition, we experimentally fitted covariates that represented different groupings for resource availability, which included (10) all cultivated food combined, (11) availability of ripe oil palm fruit and (12) availability of the other ten wild fruits. We separated oil palm fruit from the rest of the wild fruits because of the high prevalence of oil palms across the landscape, resulting in oil palm fruit availability values disproportionally large compared to other species (Supplementary Fig. S3). We measured food availability using the formula above, using mean density and DBH values calculated around each camera trap sampling site and *F* values from each phenological survey, which corresponded to each sampling period in the dataset.

### Spatiotemporal model

We employed Bayesian spatiotemporal modelling using the INLA algorithm^84,85^. For the Bayesian inference, we fitted a GMRF autoregressive spatiotemporal model with a Stochastic Partial Differential Equations (SPDE) approach^84–87^. This model accounts for spatial and temporal dependencies—a common occurrence in natural processes—via a triangulation of the study area using SPDE^85,87^. Since it was proposed eleven years ago^84^, INLA has become popular in spatial and spatiotemporal Bayesian modelling^86,88–90^. However, to our knowledge, INLA has yet to be applied to camera trap monitoring data. Following Cameletti et al. (2013), our model was based on the equation:

$$y\left({s}_{i}, t\right) = {{z}}\left({s}_{i}, t\right)\beta + \xi \left({s}_{i}, t\right)+ \varepsilon ({s}_{i}, t)$$

where $y\left({s}_{i}, t\right)$ is the spatiotemporal process that represents the Gaussian Field (GF), which in this case is the distribution of chimpanzee intensity of space use measured by the relative chimpanzee detection frequency at camera trap sampling site ${s}_{i}$ = 1…, 21 during sampling occasion $t$ = 1, …, 24. The vector of $p$ covariates is written as ${{z}}\left({s}_{i}, t\right)={({{z}}}_{1}\left({s}_{i}, t\right),\dots ,{{{z}}}_{p}\left({s}_{i}, t\right))$. $\beta = {(\beta }_{1}, \dots , {\beta }_{p})$ represents the vector of the covariate coefficients. In addition, $\xi \left({s}_{i}, t\right)$ is the spatiotemporal GF that evolves over time following a first order autoregressive process AR(1) with coefficient *a.* Lastly, $\varepsilon ({s}_{i}, t)$ is the measurement error, also referred to as nugget effect^91^.

All models were run using the *inla* function in the R package R-INLA (http://www.r-inla.org)^85,92^. Response variables were log(x + 1) transformed and all covariates were mean centred and scaled (using a z-transformation) prior to analysis. Due to occasional camera malfunctions, some NA’s were generated in the dataset for human and chimpanzee detections. At the time of this study, INLA was not able to deal with missing covariates (http://www.r-inla.org/faq#TOC-Can-INLA-deal-with-missing-covariates-). As we intended to use human detections as a covariate in the chimpanzee models, we first fitted GMRF spatiotemporal models using human detections as response variable and considered three covariates as possible predictor for human site use (minimum distance to village, agriculture and road). We extracted the final model’s posterior means of the measured spatiotemporal field, which corresponded to the mean estimates measured at the 21 sampling sites at each sampling occasion (N = 24), totalling 504 values of which 51 were newly generated values from the missing observations. The posterior means and the corresponding observed data were highly correlated (*r* = 0.95, N = 453, *P* < 0.00001) and were thus deemed suitable to be included as a covariate in the chimpanzee models. Posterior results including the hyperparameters of the human spatiotemporal model are available in Supplementary Tables S3–S4. A default Gaussian prior was used for all models^86^. Model selection was based on the Deviance Information Criterion (DIC)^85^. We ran a chimpanzee model without covariates and a model including all covariates considered (Supplementary Table S5). We gradually excluded covariates in a stepwise procedure to identify the model with the lowest DIC. Model fit was based on the DIC value and the posterior density symmetry of the parameters showed by the equal mean, credible interval quantiles at 50% and the mode presented in Table 1 and showed in Supplementary Fig. S4^93^. The predictive power of the covariates was determined based on the 95% credible interval not overlapping with zero. See the Supplementary Information for additional description on the spatiotemporal model structure including the triangulation mesh of the study area (Supplementary Fig. S1) and a summary of the hyperparameters of the final model (Supplementary Table S6). All analyses were ran on R version 4.0.2^94^.

## Supplementary Information

Supplementary Information 1.

Supplementary Video 1.

## Data Availability

The datasets generated for this study are available from the corresponding author on request. Camera trap data may be requested in a record table form.

## References

[1] Dirzo R (2014). Defaunation in the anthropocene. Science.

[2] Boivin NL (2016). Ecological consequences of human niche construction: examining long-term anthropogenic shaping of global species distributions. Proc. Natl. Acad. Sci..

[3] Newbold T (2015). Global effects of land use on local terrestrial biodiversity. Nature.

[4] Hagen M, Jacob U, Woodward G (2012). Biodiversity, species interactions and ecological networks in a fragmented world. Advances in Ecological Research.

[5] Gallego-Zamorano J (2020). Combined effects of land use and hunting on distributions of tropical mammals. Conserv. Biol..

[6] Ellis EC, Ramankutty N (2008). Putting people in the map: anthropogenic biomes of the world. Front. Ecol. Environ..

[7] Estrada A, Raboy BE, Oliveira LC (2012). Agroecosystems and primate conservation in the tropics: a review. Am. J. Primatol..

[8] Bhagwat SA, Willis KJ, Birks HJB, Whittaker RJ (2008). Agroforestry: a refuge for tropical biodiversity?. Trends Ecol. Evol..

[9] Galán-Acedo C (2019). The conservation value of human-modified landscapes for the world’s primates. Nat. Commun..

[10] Arroyo-Rodríguez V (2020). Designing optimal human-modified landscapes for forest biodiversity conservation. Ecol. Lett..

[11] Kshettry A, Vaidyanathan S, Sukumar R, Athreya V (2020). Looking beyond protected areas: identifying conservation compatible landscapes in agro-forest mosaics in north-eastern India. Glob. Ecol. Conserv..

[12] Osborn, F. V. & Hill, C. M. Techiques to reduce crop loss: human and technical dimensions in Africa. In *People and Wildlife, Conflict or Co-existence?* 72–85 (Cambridge University Press, Cambridge, 2005).

[13] McLennan MR, Asiimwe C (2016). Cars kill chimpanzees: case report of a wild chimpanzee killed on a road at Bulindi, Uganda. Primates J. Primatol..

[14] Chapman CA (2006). Do food availability, parasitism, and stress have synergistic effects on red colobus populations living in forest fragments?. Am. J. Phys. Anthropol..

[15] Goldberg TL, Gillespie TR, Rwego IB, Estoff EL, Chapman CA (2008). Forest fragmentation as cause of bacterial transmission among nonhuman primates, humans, and livestock, Uganda. Emerg. Infect. Dis..

[16] McLennan MR, Hyeroba D, Asiimwe C, Reynolds V, Wallis J (2012). Chimpanzees in mantraps: lethal crop protection and conservation in Uganda. Oryx.

[17] Kalema-Zikusoka, G., Rubanga, S., Mutahunga, B. & Sadler, R. Prevention of Cryptosporidium and GIARDIA at the human/gorilla/livestock interface. *Front. Public Health* **6**, (2018). 10.3389/fpubh.2018.00364 PMC6302101 30619805

[18] Kenney J, Allendorf FW, McDougal C, Smith JLD (2014). How much gene flow is needed to avoid inbreeding depression in wild tiger populations?. Proc. R. Soc. B Biol. Sci..

[19] Willems EP, Hill RA (2009). Predator-specific landscapes of fear and resource distribution: effects on spatial range use. Ecology.

[20] Coleman BT, Hill RA (2014). Living in a landscape of fear: the impact of predation, resource availability and habitat structure on primate range use. Anim. Behav..

[21] Palmer MS, Fieberg J, Swanson A, Kosmala M, Packer C (2017). A ‘dynamic’ landscape of fear: prey responses to spatiotemporal variations in predation risk across the lunar cycle. Ecol. Lett..

[22] Laundré, J. W., Hernandez, L. & Ripple, W. J. The landscape of fear: ecological implications of being afraid. *Open Ecol. J.* **3**, (2010).

[23] Theuerkauf J, Rouys S (2008). Habitat selection by ungulates in relation to predation risk by wolves and humans in the Białowieża Forest, Poland. For. Ecol. Manag..

[24] Ciuti S (2012). Effects of humans on behaviour of wildlife exceed those of natural predators in a landscape of fear. PLoS ONE.

[25] Nowak K, Wimberger K, Richards SA, Hill RA, le Roux A (2017). Samango monkeys (*Cercopithecus albogularis labiatus*) manage risk in a highly seasonal, human-modified landscape in Amathole Mountains, South Africa. Int. J. Primatol..

[26] Suraci JP, Clinchy M, Zanette LY, Wilmers CC (2019). Fear of humans as apex predators has landscape-scale impacts from mountain lions to mice. Ecol. Lett..

[27] Carter NH, Shrestha BK, Karki JB, Pradhan NMB, Liu J (2012). Coexistence between wildlife and humans at fine spatial scales. Proc. Natl. Acad. Sci..

[28] Carter NH, Jasny M, Gurung B, Liu J (2015). Impacts of people and tigers on leopard spatiotemporal activity patterns in a global biodiversity hotspot. Glob. Ecol. Conserv..

[29] Lamb CT (2020). The ecology of human–carnivore coexistence. Proc. Natl. Acad. Sci..

[30] Bryson-Morrison N, Tzanopoulos J, Matsuzawa T, Humle T (2017). Activity and habitat use of chimpanzees (*Pan troglodytes verus*) in the anthropogenic landscape of Bossou, Guinea, West Africa. Int. J. Primatol..

[31] de Almeida-Rocha JM, Peres CA, Oliveira LC (2017). Primate responses to anthropogenic habitat disturbance: a pantropical meta-analysis. Biol. Conserv..

[32] Galán-Acedo C, Arroyo-Rodríguez V, Cudney-Valenzuela SJ, Fahrig L (2019). A global assessment of primate responses to landscape structure. Biol. Rev..

[33] Garriga RM (2019). Factors influencing wild chimpanzee (*Pan troglodytes verus*) relative abundance in an agriculture-swamp matrix outside protected areas. PLoS ONE.

[34] Hockings KJ, Anderson JR, Matsuzawa T (2006). Road crossing in chimpanzees: a risky business. Curr. Biol..

[35] Estrada A (2017). Impending extinction crisis of the world’s primates: why primates matter. Sci. Adv..

[36] IUCN SSC Primate Specialist Group. *Regional action plan for the conservation of western chimpanzees (*Pan troglodytes verus*) 2020–2030.* (2020).

[37] Kalan AK (2020). Environmental variability supports chimpanzee behavioural diversity. Nat. Commun..

[38] Hockings KJ, Anderson JR, Matsuzawa T (2012). Socioecological adaptations by chimpanzees, *Pan troglodytes verus*, inhabiting an anthropogenically impacted habitat. Anim. Behav..

[39] McLennan MR, Hockings KJ (2014). Wild chimpanzees show group differences in selection of agricultural crops. Sci. Rep..

[40] Kalan AK (2019). Novelty response of wild African apes to camera traps. Curr. Biol..

[41] Hockings KJ, McLennan MR (2012). From forest to farm: systematic review of cultivar feeding by chimpanzees—management implications for wildlife in anthropogenic landscapes. PLoS ONE.

[42] Hockings KJ, Anderson JR, Matsuzawa T (2009). Use of wild and cultivated foods by chimpanzees at Bossou, Republic of Guinea: feeding dynamics in a human-influenced environment. Am. J. Primatol..

[43] McLennan MR (2013). Diet and feeding ecology of chimpanzees (*Pan troglodytes*) in Bulindi, Uganda: foraging strategies at the forest–farm interface. Int. J. Primatol..

[44] McLennan MR, Ganzhorn JU (2017). Nutritional characteristics of wild and cultivated foods for chimpanzees (*Pan troglodytes*) in agricultural landscapes. Int. J. Primatol..

[45] Matthews A, Matthews A (2004). Survey of gorillas (*Gorilla gorilla gorilla*) and chimpanzees (*Pan troglodytes troglodytes*) in Southwestern Cameroon. Primates.

[46] Morgan D (2018). African apes coexisting with logging: comparing chimpanzee (*Pan troglodytes troglodytes*) and gorilla (*Gorilla gorilla gorilla*) resource needs and responses to forestry activities. Biol. Conserv..

[47] Krief S (2014). Wild chimpanzees on the edge: nocturnal activities in croplands. PLoS ONE.

[48] Riley EP, Priston NEC (2010). Macaques in farms and folklore: exploring the human–nonhuman primate interface in Sulawesi, Indonesia. Am. J. Primatol..

[49] Parathian HE, McLennan MR, Hill CM, Frazão-Moreira A, Hockings KJ (2018). Breaking through disciplinary barriers: human–wildlife interactions and multispecies ethnography. Int. J. Primatol..

[50] Fuentes A, Gamerl S (2005). Disproportionate participation by age/sex classes in aggressive interactions between long-tailed macaques (*Macaca fascicularis*) and human tourists at Padangtegal monkey forest, Bali, Indonesia. Am. J. Primatol..

[51] McLennan, M. R. & Hockings, K. J. The aggressive apes? Causes and contexts of great ape attacks on local persons. In *Problematic Wildlife* (ed. Angelici, F. M.) 373–394 (Springer, Cham, 2016). 10.1007/978-3-319-22246-2_18.

[52] Hill CM, Webber AD (2010). Perceptions of nonhuman primates in human–wildlife conflict scenarios. Am. J. Primatol..

[53] McLennan MR, Hill CM (2012). Troublesome neighbours: changing attitudes towards chimpanzees (Pan troglodytes) in a human-dominated landscape in Uganda. J. Nat. Conserv..

[54] Mito Y, Sprague DS, Radhakrishna S, Huffman MA, Sinha A (2013). The Japanese and Japanese monkeys: dissonant neighbors seeking accommodation in a shared habitat. The Macaque Connection: Cooperation and Conflict Between Humans and Macaques.

[55] Morzillo, A., de Beurs, K. & Martin-Mikle, C. A conceptual framework to evaluate human-wildlife interactions within coupled human and natural systems. *Ecol. Soc.* **19**, (2014).

[56] Martin J (2010). Coping with human disturbance: spatial and temporal tactics of the brown bear (*Ursus arctos*). Can. J. Zool..

[57] Hockings KJ (2007). Chimpanzees share forbidden fruit. PLoS ONE.

[58] Duvall CS (2008). Human settlement ecology and chimpanzee habitat selection in Mali. Landsc. Ecol..

[59] Hockings, K. J., Parathian, H., Bessa, J. & Frazão-Moreira, A. Extensive overlap in the selection of wild fruits by chimpanzees and humans: implications for the management of complex social-ecological systems. *Front. Ecol. Evol.* **8**, (2020).

[60] Nowak K, Hill RA, Wimberger K, le Roux A, Waller MT (2016). Risk-taking in samango monkeys in relation to humans at two sites in South Africa. Ethnoprimatology: Primate Conservation in the 21st Century.

[61] INE. *Recenseamento Geral da População e Habitação: População por Região, Sector e Localidades por Sexo Censo 2009*. 160 (2009).

[62] Heinicke, S. *et al.* Characteristics of positive deviants in western chimpanzee populations. *Front. Ecol. Evol.* **7**, (2019).

[63] Bersacola E (2020). Zooming in on Human-Wildlife Coexistence: Primate Community Responses in a Shared Agroforest Landscape in Guinea-Bissau.

[64] Bessa J, Sousa C, Hockings KJ (2015). Feeding ecology of chimpanzees (*Pan troglodytes verus*) inhabiting a forest-mangrove-savanna-agricultural matrix at Caiquene-Cadique, Cantanhez National Park, Guinea-Bissau. Am. J. Primatol..

[65] Hockings, K. J. *et al.* Leprosy in wild chimpanzees. *bioRxiv* 2020.11.10.374371 (2020) 10.1101/2020.11.10.374371.

[66] Hockings KJ, Sousa C (2012). Differential utilization of cashew—a low-conflict crop—by sympatric humans and chimpanzees. Oryx.

[67] Calenge C (2006). The package adehabitat for the R software: a tool for the analysis of space and habitat use by animals. Ecol. Model..

[68] Schmid F, Schmidt A (2006). Nonparametric estimation of the coefficient of overlapping—theory and empirical application. Comput. Stat. Data Anal..

[69] Ridout MS, Linkie M (2009). Estimating overlap of daily activity patterns from camera trap data. J. Agric. Biol. Environ. Stat..

[70] Hijmans, R. J. *Raster: geographic data analysis and modeling.* (2020).

[71] Khorozyan I, Stanton D, Mohammed M, Al-Rail W, Pittet M (2014). Patterns of co-existence between humans and mammals in Yemen: some species thrive while others are nearly extinct. Biodivers. Conserv..

[72] Sousa J, Barata AV, Sousa C, Casanova CCN, Vicente L (2011). Chimpanzee oil-palm use in southern Cantanhez National Park, Guinea-Bissau. Am. J. Primatol..

[73] Tutin CEG (1991). Foraging profiles of sympatric lowland gorillas and chimpanzees in the Lope Reserve, Gabon [and discussion]. Philos. Trans. Biol. Sci..

[74] Yamakoshi G (1998). Dietary responses to fruit scarcity of wild chimpanzees at Bossou, Guinea: possible implications for ecological importance of tool use. Am. J. Phys. Anthropol..

[75] Wilson ML, Hauser MD, Wrangham RW (2007). Chimpanzees (*Pan troglodytes*) modify grouping and vocal behaviour in response to location-specific risk. Behaviour.

[76] Lindshield S, Danielson BJ, Rothman JM, Pruetz JD (2017). Feeding in fear? How adult male western chimpanzees (*Pan troglodytes verus*) adjust to predation and savanna habitat pressures. Am. J. Phys. Anthropol..

[77] Olson DM (2001). Terrestrial ecoregions of the world: a new map of life on Earth. Bioscience.

[78] Sousa J, Vicente L, Gippoliti S, Casanova C, Sousa C (2014). Local knowledge and perceptions of chimpanzees in Cantanhez National Park, Guinea-Bissau. Am. J. Primatol..

[79] Sharma K (2020). Conservation and people: towards an ethical code of conduct for the use of camera traps in wildlife research. Ecol. Solut. Evid..

[80] Sun C (1996). Tree phenology in a tropical montane forest in Rwanda. Biotropica.

[81] McLennan MR (2010). Chimpanzee ecology and interactions with people in an unprotected human-dominated landscape at Bulindi, Western Uganda.

[82] Jenks KE (2011). Using relative abundance indices from camera-trapping to test wildlife conservation hypotheses—an example from Khao Yai National Park, Thailand. Trop. Conserv. Sci..

[83] O’Brien TG, Kinnaird MF, Wibisono HT (2003). Crouching tigers, hidden prey: sumatran tiger and prey populations in a tropical forest landscape. Anim. Conserv. Forum.

[84] Rue H, Martino S, Chopin N (2009). Approximate Bayesian inference for latent Gaussian models by using integrated nested Laplace approximations. *J. R*. Stat. Soc. Ser. B Stat. Methodol..

[85] Blangiardo M, Cameletti M, Baio G, Rue H (2013). Spatial and spatio-temporal models with R-INLA. Spat. Spatio-Temporal Epidemiol..

[86] Cameletti M, Lindgren F, Simpson D, Rue H (2013). Spatio-temporal modeling of particulate matter concentration through the SPDE approach. AStA Adv. Stat. Anal..

[87] Lindgren F, Rue H, Lindström J (2011). An explicit link between Gaussian fields and Gaussian Markov random fields: the stochastic partial differential equation approach. J. R Stat. Soc. Ser. B Stat. Methodol..

[88] Bakka H (2018). Spatial modeling with R-INLA: a review. Wiley Interdiscip. Rev. Comput. Stat..

[89] Noor AM (2014). The changing risk of *Plasmodium falciparum* malaria infection in Africa: 2000–10: a spatial and temporal analysis of transmission intensity. Lancet.

[90] Rue H (2017). Bayesian computing with INLA: a review. Annu. Rev. Stat. Its Appl..

[91] Cressie N, Wikle CK (2015). Statistics for Spatio-Temporal Data.

[92] Lindgren F, Rue H (2015). Bayesian spatial modelling with R-INLA. J. Stat. Softw..

[93] Spiegelhalter DJ, Best NG, Carlin BP, Linde AVD (2002). Bayesian measures of model complexity and fit. J. R. Stat. Soc. Ser. B Stat. Methodol..

[94] R Core Team. *R: A language and environment for statistical computing*. (R Foundation for Statistical Computing, 2020).

