# Supplementary material for: Chimpanzees balance resources and risk in an anthropogenic landscape of fear
Source: Sci Rep. 2021 Feb 25;11:4569. doi: 10.1038/s41598-021-83852-3 (PMC7907193; doi:10.1038/s41598-021-83852-3)
Supplement: Supplementary file 1 — Supplementary Information 1. [file 41598_2021_83852_MOESM1_ESM.docx]

Supplementary Information

Chimpanzees balance resources and risk in an anthropogenic landscape of fear

Elena Bersacola*^1,2,3^, Catherine M. Hill^2^ and Kimberley J. Hockings^1,3^

^1^ Centre for Ecology and Conservation, University of Exeter, Penryn, Cornwall, UK

^2^ Department of Social Sciences, Oxford Brookes University, Oxford, UK

^3^ Centre for Research in Anthropology (CRIA), Lisbon, Portugal

*Corresponding author

Email: [e.bersacola@exeter.ac.uk](mailto:xxxxx@xxxx.xxx)

**This PDF file includes:**

Supplementary text

Figures S1 to S4

Tables S1 to S6

Legend for Movie S1

SI References

**Additional Supporting Information materials for this manuscript include:**

Movie S1

**Materials and Methods**

Additional information on covariates used in the spatiotemporal model

The spatial and spatiotemporal covariates used in the INLA spatiotemporal model are presented in Supplementary Table S2. A ‘village’ was defined as a permanent human settlement comprising at least ten households. Thus, based on previous censuses^1,2^, within the spatial range of this study’s chimpanzee community we included three villages, namely Cadique Nalu (29 households), Caiquene (15 households) and Cadique Iala (>15 households). We defined ‘roads’ as those that are accessible by car. Within our study area roads were relatively small and, in some areas, they resembled more of a large footpath. At the time of the study the roads were not tarmacked, and vehicle traffic mainly comprised people on foot or bicycle, and occasionally motorbike. The two roads included in this study were the road that runs from Iemberem to Cadique Nalu and its intersecting road to Caiquene. As chimpanzees will behave differently according to the level of exposure in human land uses, we first distinguished between types of agriculture that have distinctive vegetation cover, i.e. shifting cultivation fields (*lugares* in Guinea-Bissau Creole language) and mangroves rice fields versus orchards. However, the variables ‘distance to orchard’ and ‘distance to cultivated field/mangrove rice’ were highly correlated (*r* = 0.90, N = 21, *P* < 0.00001). We therefore merged the two covariates into one (dist_agriculture), where the agricultural area nearest to camera trap sites included orchard (N sites = 15), shifting cultivation field (N sites = 5) and mangrove rice field (N sites = 1), bearing in mind that most cashew orchards were near cultivated fields and vice versa. We define the ‘forest block’ as the continuous protected forest located south of the Iemberem-Cadique road and west of the Caiquene road (see Fig. 1 in main text). If camera trap sites were located within the landscape feature(s), i.e. village/road/agriculture/forest block, we assigned a zero value for the Euclidean distance (i.e. 0 m). For sites located outside, we measured the Euclidean distance to the edge of the feature. Human detection frequencies (human_det) were derived from the human spatiotemporal model estimated values across the 21 camera traps and 24 sampling periods (see Supplementary Table S3). The covariates used to measure the spatiotemporal variation in food availability included the availability of wild ripe fruit (covariate wild_fruit which included all eleven species, or covariates oil_palm_fruit and ten_wild_fruits considered separately) and the availability of six cultivated fruits (cultivated_fruit when considered togheter). Depending on the type and location, cultivated foods are associated with different levels of risk. For example, cashew orchards are present throughout the landscape and chimpanzees only feed on the pseudo fruit leaving the commercially valuable nut intact^3^. In contrast, orange trees are fewer, require high maintenance and the fruit, which is consumed by chimpanzees, is economically valuable to farmers^4^. Farmers keep orange orchards at the villages to ease maintenance and harvest. In addition, cultivated foods only present in villages (orange, lime and papaya) are available when wild fruits are scarce. To account for the spatial risk difference and test for hypothesis 3 (evidence for temporal risk trade-off in favour of optimal foraging), we separated cultivated fruits into three groups: those present across much of the heterogeneous matrix (cashew), those present in both villages and abandoned villages (mango_baobab) and those present in villages only, available when wild fruits are scarce (orange_lime_papaya). Overall monthly food availability index (FAI) of ripe fruit of each plant species are shown in Supplementary Fig. S2–S3.

Additional information on the spatiotemporal model structure

Our modelling approach followed Cameletti et al (2013). A random field consists of spatially georeferenced numbers that define a process. The Gaussian Field (GF) is the random field and its interaction with variables, i.e. the covariance function, that varies through space and time^5^. For example, in this study the random field is represented by the variation in intensity of space use by chimpanzees within their home range, and the GF also involves the relationship of chimpanzee movements with the covariates (e.g. risk, resources). The GF is termed “Gaussian” because it refers to the Gaussian probability density functions of the covariates. One of the key aspects of any spatiotemporal processes that must be addressed in spatiotemporal modelling is that of autocorrelation. In the random field, the spatially georeferenced numbers are often assumed to be correlated in space and time, or to covary. In other words, measurements at neighbouring points are more related to one another than those at more distant points, and measurements taken at consecutive time points are more likely to depend on one another than time points measured with a larger time gap. The Matérn covariance function is a widely used approach to describe how the covariance between two measurements changes with their distance apart. Certain GFs with a Matérn covariance function can be represented as GMRFs, with the GMRF constructed using a particular SPDE^6^. Representing a GF as a GMRF enables it to be described using a sparse matrix and considerably reduces the computational time required to estimate the covariance structure of the model. GMRF (also known as conditional auto-regressions [CAR]) are already commonly used in spatiotemporal analysis, and the reduction in computation time generated by incorporating the SPDE approach makes it possible to include more fine-scale spatial data over larger areas.

Using INLA, through the R package R-INLA, it is possible to fit spatiotemporal models incorporating the SPDE approach^7^. The triangulation mesh used in the SPDE approach is built using the R-INLA package function *inla.mesh.create.helper*, providing the study area sampling points and borders, and indicating the Euclidean distance of the limits from the study area borders, the absolute outer limits (to decrease edge effect), and their corresponding maximal edge lengths and minimal allowed vertice angles^8,9^. Supplementary Fig. S1 shows the triangulation mesh we created for the Caiquene–Cadique study area. The mesh is then incorporated in the SPDE and the data structure necessary to run the model. The R-INLA model results provide the posterior estimates of the latent random field, the beta coefficients of the covariates and the hyperparameters. The hyperparameters consist of the measurement error and the spatiotemporal dependencies given to the latent field. The hyperparameters are the following:

$\sigma_{\varepsilon}^{2}$ = measurement error variance: also referred to as nugget effect ^10^. The measurement error corresponds to the difference between the observed value and the true value, i.e. a residual variance that is spatially and temporally uncorrelated.

$\sigma_{\omega}^{2}$ = the marginal variance of the GMRF^6^;

*a* = temporal autocorrelation coefficient of the AR(1);

*ρ* = empirically-derived spatial autocorrelation: the Euclidean distance where the spatial correlation is close to null, i.e. where there is no more spatial correlation.

Cameletti et al. (2013) and Lindgen et al. (2011) provide detailed information on the concepts behind this type of model, including all relevant equations. We used R codes provided in Cameletti et al. (2013).

Extended technical description of results

Description of the hyperparameters of the selected chimpanzee model

The hyperparameters (Supplementary Table S6) of the selected spatiotemporal model for chimpanzee intensity of space use show that the GMRF variance $\sigma_{\omega}^{2}$ explains more of the spatial variation compared to the residual measurement error $\sigma_{\varepsilon}^{2}$, therefore indicating that the spatial field is important. The empirically derived spatial correlation value *ρ* shows that at a distance of 495 m the correlation is close to null, revealing that the spatial extent of the correlation was low. The minimum distance between camera trap sites was 520 m, a strong correlation between sampling sites was therefore unlikely. The value of the temporal correlation coefficient *a* indicates high temporal correlation, showing that in general, changes in intensity of space use by chimpanzees evolved relatively slowly over time (i.e. chimpanzee tended to use sites at similar frequencies for longer than two weeks).

*
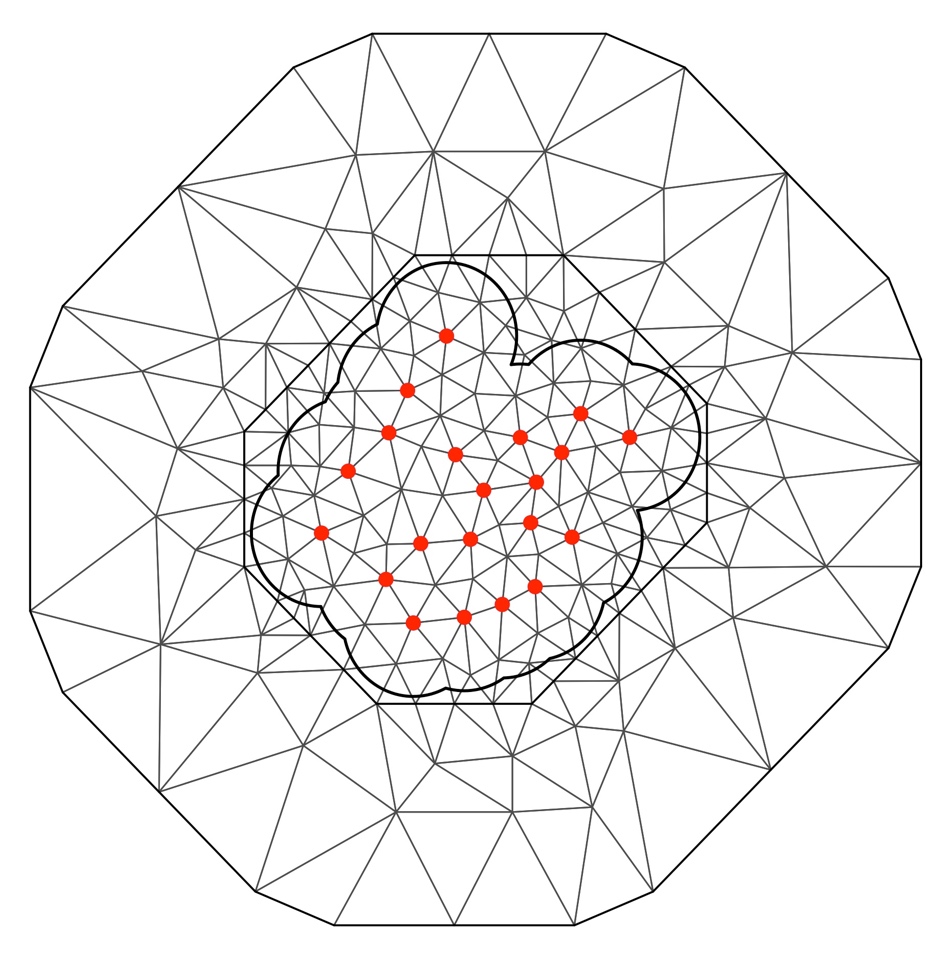
*

**Figure S1.** Triangulation mesh of the study area using 192 vertices. Red circles represent the 21 camera trap sampling sites. This figure was created using the *inla.mesh.create.helper* function in the R package R-INLA^8,11^.


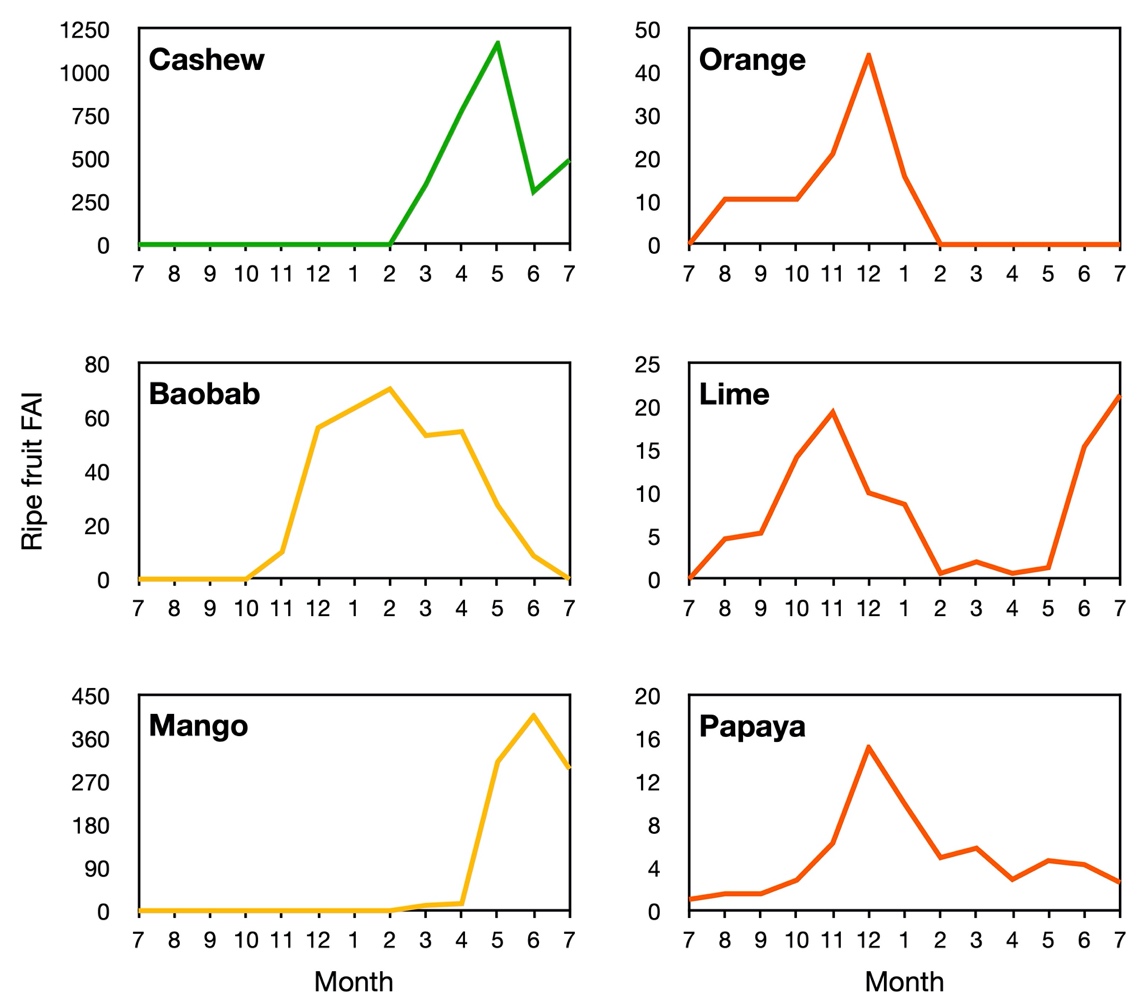


**Figure S2.** Monthly food availability index (FAI) of cultivated ripe fruits measured at Caiquene–Cadique during our study period.


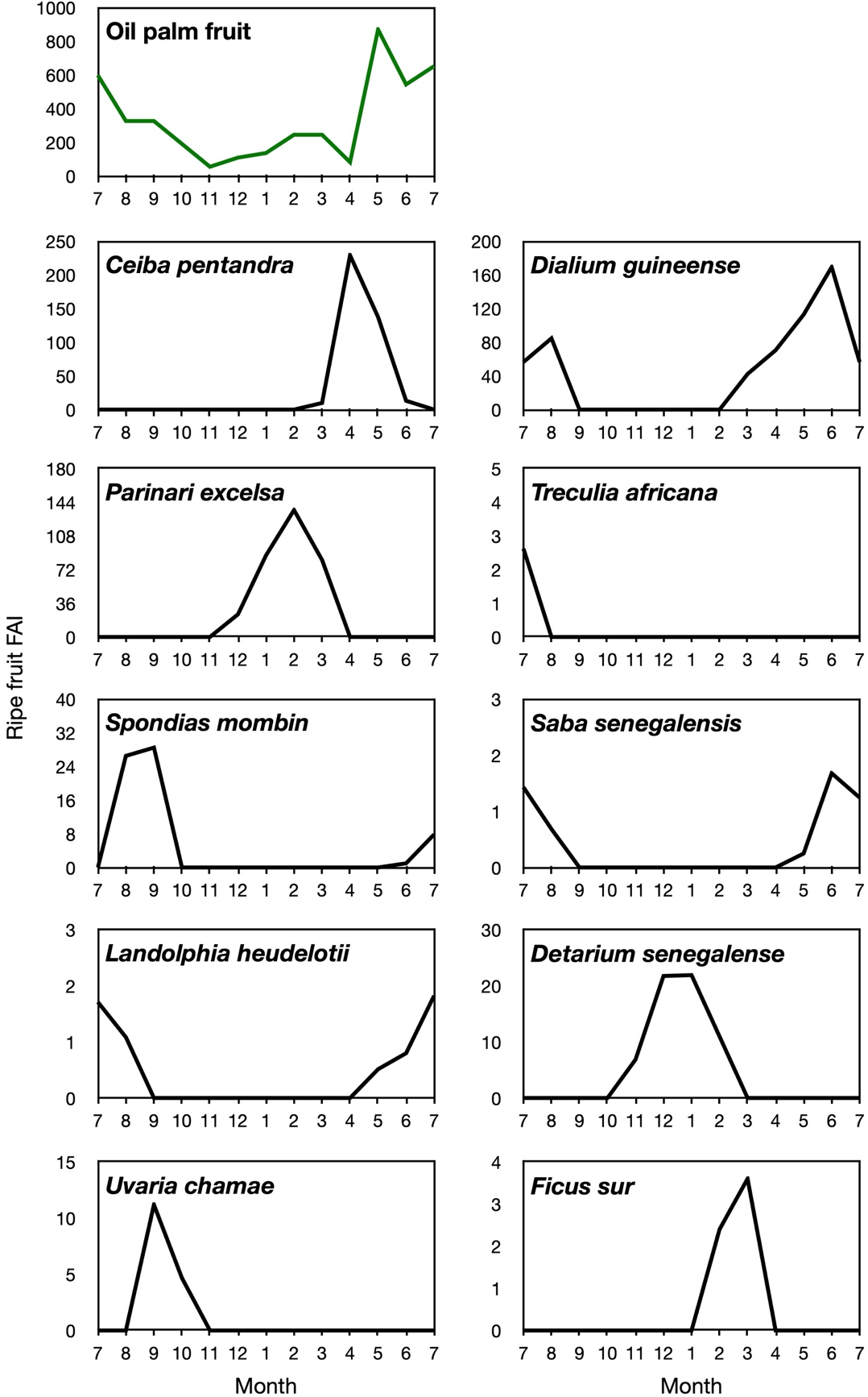


**Figure S3.** Monthly food availability index (FAI) of wild ripe fruits measured at Caiquene–Cadique during our study period.

**
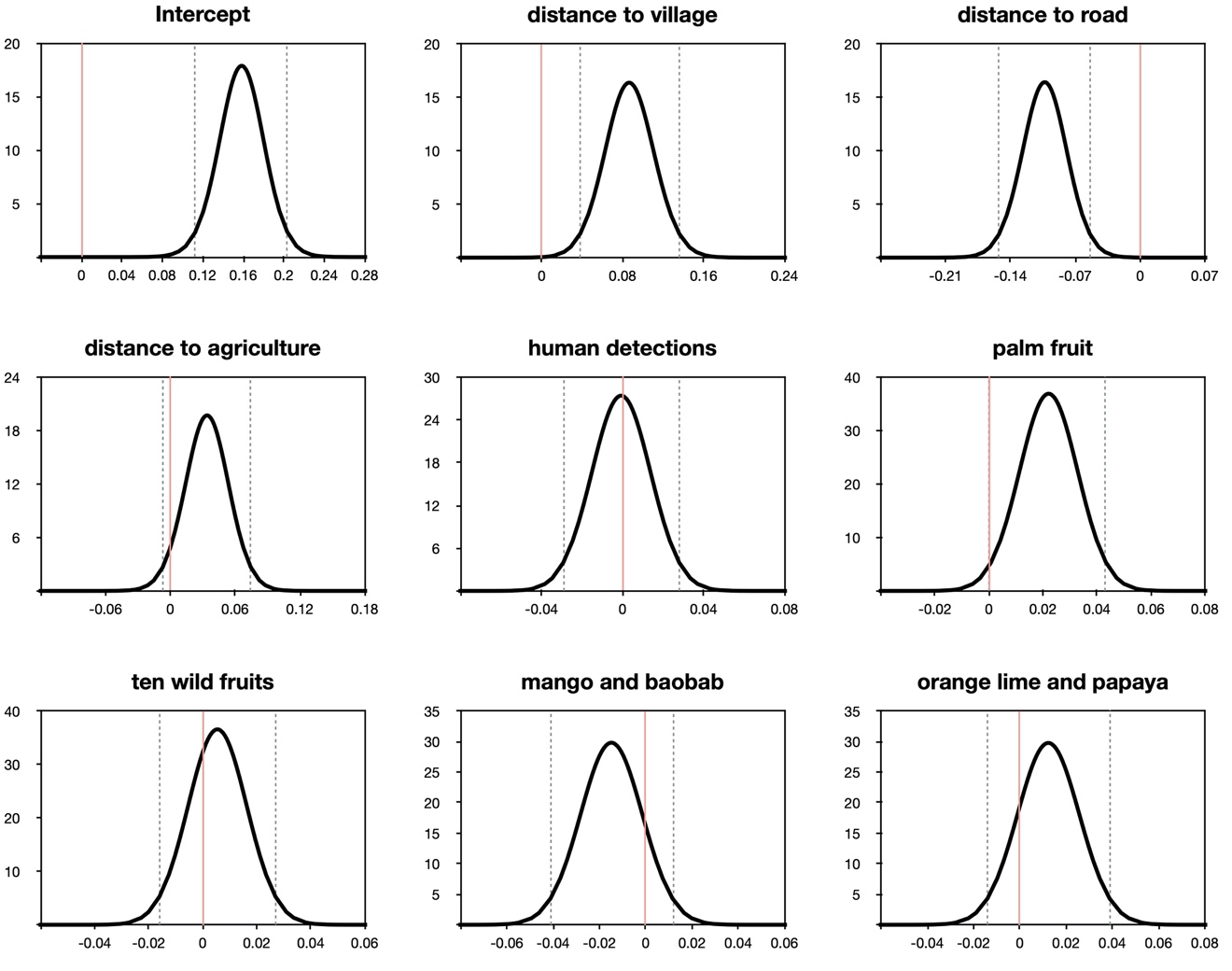
**

**Figure S4.** Marginal posterior density plots for the fixed effects of the model parameters. Pink line represents the zero value and dashed grey lines show 0.025 and 0.975 quantiles of credible intervals. All posterior density means show symmetrical densities and align with the credible interval quantiles at 50% indicating adequate fit between the best model’s parameters and the GMRF.

Table S1. Chimpanzee food tree species (11 wild and 6 cultivated) monitored for phenology. Species selection was based on feeding ecology data collected in Caiquene–Cadique in 2013^1,12^. Density of adult trees was calculated from 453 plots.

| Family | Species | Type | N of individuals  monitored | Min. DBH adults (cm)^a^ | Mean DBH monitored (cm) | Density in plots (stems/ha) | Mean DBH in plots (cm) | Local name  (Guinea-Bissau Creole) |
| --- | --- | --- | --- | --- | --- | --- | --- | --- |
| Palmae | *Elaeis guineensis* | wild | 10 | na^b^ | 42.42 (±6.44) | 14.46 | 37.54 (±11.31) | *Palmeira* |
| Leguminosae | *Dialium guineense* | wild | 10 | 10 | 50.61 (±26.14) | 12.25 | 23.01 (±13.83) | *Veludo* |
| Bombacaceae | *Ceiba pentandra* | wild | 10 | 30 | 133.31 (±49.47) | 0.99 | 66.05 (±34.64) | *Poilon* |
| Moraceae | *Treculia africana* | wild | 10 | 20 | 64.87 (±22.49) | 0.77 | 34.06 (±11.88) | *Mantchambe* |
| Chrysobalanaceae | *Parinari excelsa* | wild | 10 | 20 | 89.17 (±21.42) | 1.77 | 54.71 (±32.78) | *Mampataz* |
| Apocynaceae | *Saba senegalensis* | wild | 10 | 10^c^ | 8.66 (±5.27) | 0.11 | 11.3 | *Foli elefante* |
| Anacardiaceae | *Spondias mombin* | wild | 10 | 10 | 44.30 (±20.35) | 1.21 | 16.22 (±4.94) | *Mandipli* |
| Leguminosae | *Detarium senegalense* | wild | 10 | 15 | 43.69 (±18.84) | 0.55 | 49.72 (±32.57) | *Mambodi* |
| Apocynaceae | *Landolphia heudelotii* | wild | 10 | 10^c^ | 5.06 (±1.56) | 0.11 | 10.35 | *Foli macaco* |
| Moraceae | *Ficus sur* | wild | 10 | 20 | 58.34 (±21.63) | 0.77 | 31.19 (±5.45) | *Figuera tonkinjá* |
| Annonaceae | *Uvaria chamae* | wild | 10 | 10^c^ | 5.45 (±1.53) | 1.43 | 13.05 (±1.83) | *Banana santchu* |
| Anacardiaceae | *Anacardium occidentale* | cult. | 10 | 10 | 22.99 (± 7.77) | 21.96 | 18.61 (±6.39) | *Cadjú* |
| Bombacaceae | *Adansonia digitata* | cult. | 8 | 60 | 105.89 (±14.07) | 0.22 | 104.72 (±34.21) | *Cabaceira* |
| Anacardiaceae | *Mangifera indica* | cult. | 20 | 20 | 87.25 (±28.70) | 2.21 | 66.92 (±29.80) | *Mango* |
| Rutaceae | *Citrus sinensis* | cult. | 10 | 15 | 36.75 (±10.80) | 1.21 | 28.94 (±13.48) | *Limão de tera* |
| Rutaceae | *Citrus aurantifolia* | cult. | 10 | 4 | 18.98 (±10.90) | 0.66 | 20.21 (±18.78) | *Larandja* |
| Caricaceae | *Carica papaya* | cult. | 10 | 10 | 21.43 (±4.26) | 0.44 | 23.71 (±3.87) | *Papaya* |

^a^ Taken from^13–15^ and based on personal observations during fieldwork.

^b^ Minimum adult palm tree based on height >5 m due to younger palms having larger DBH compared to adult trees.

^c^ Measured equal or above the reported DBH but smaller stems can also fruit.

**Table S2.** Covariates considered in the chimpanzee spatiotemporal models. Spatial covariates vary across space (21 camera trap sites) and remain constant in time. Spatiotemporal covariates differ across both space (21 camera trap sites) and time (24 sampling periods).

| # | Model covariates | Code | Variation |  |
| --- | --- | --- | --- | --- |
| 1) | Distance to village | dist_village | Spatial | Risk |
| 2) | Distance to road | dist_road | Spatial | Risk |
| 3) | Distance to agriculture | dist_agriculture | Spatial | Risk |
| 4) | Distance to forest block | dist_forest | Spatial | Risk |
| 5) | Human detections | human_det | Spatiotemporal | Risk |
| 6) | Wild fruit | wild_fruit | Spatiotemporal | Resource |
| 7) | Cashew | cashew | Spatiotemporal | Low risk resource |
| 8) | Mango and baobab | mango_baobab | Spatiotemporal | Medium risk resources |
| 9) | Orange lime and papaya | orange_lime_papaya | Spatiotemporal | High risk resources |
| 10) | All cultivated fruits | cultivated_fruit | Spatiotemporal | Resource |
| 11) | Oil palm fruit | palm_fruit | Spatiotemporal | Resource |
| 12) | Ten wild fruits | ten_wild_fruits | Spatiotemporal | Resource |

**Table S3.** Posterior means of the selected human spatiotemporal model. DIC = –628.8316, compared to no-covariate model DIC = –602.8237, ∆DIC = 26.0079.

| Covariate | Mean | ±SD | 2.5% | 50% | 97.5% | Mode |
| --- | --- | --- | --- | --- | --- | --- |
| Intercept | 0.209 | 0.030 | 0.150 | 0.209 | 0.268 | 0.209 |
| Distance to village | -0.087 | 0.029 | -0.145 | -0.088 | -0.029 | -0.088 |

**Table S4.** Posterior estimates of hyperparameters of the selected human spatiotemporal model showing means, standard deviations and credible interval quantiles.

| Parameter | Mean | ±SD | 2.5% | 50% | 97.5% |
| --- | --- | --- | --- | --- | --- |
| $\sigma_{\varepsilon}^{2}$ | 0.0082 | 0.0023 | 0.0044 | 0.0080 | 0.0133 |
| $\sigma_{\omega}^{2}$ | 0.5410 | 0.5779 | 0.0556 | 0.3610 | 2.1109 |
| *a* | 200.315 | 108.772 | 67.035 | 174.389 | 482.833 |
| *ρ* | 0.8279 | 0.0403 | 0.7341 | 0.8334 | 0.8913 |

$\sigma_{\varepsilon}^{2}$ = measurement error of variance referred to as nugget effect^10^; $\sigma_{\omega}^{2}$ = marginal variance of the Gaussian Markov Random Field^6^; *a* = temporal autocorrelation coefficient AR(1); *ρ* = empirically derived spatial autocorrelation.

**Table S5.** List of chimpanzee spatiotemporal models considered in this study. Models are listed in ascending order based on their Deviance Information Criterion (DIC). Formulae reported in this table omit spatiotemporal dependencies with R code: + f(field, model = spde, group = field.group, control.group = list(model="ar1")).

| # | Model formula | DIC |
| --- | --- | --- |
| 1 | logCHIMP_det ~ -1 + Intercept + mango_baobab + orange_lime_papaya + palm_fruit + ten_wild_fruits + dist_road + dist_agriculture + dist_village + hum_det | -737.2343 |
| 2 | logCHIMP_det ~ -1 + Intercept + cashew + mango_baobab + orange_lime_papaya + wild_fruit + dist_forest + dist_road + dist_agriculture + dist_village + hum_det | -736.2259 |
| 3 | logCHIMP_det ~ -1 + Intercept + cashew + mango_baobab + orange_lime_papaya + wild_fruit + dist_forest + dist_road + dist_agriculture + dist_village | -735.7906 |
| 4 | logCHIMP_det ~ -1 + Intercept + wild_fruit + dist_road + dist_village + dist_agriculture | -735.6095 |
| 5 | logCHIMP_det ~ -1 + Intercept + cashew + mango_baobab + orange_lime_papaya + wild_fruit + dist_road + dist_agriculture + dist_village + hum_det | -735.2871 |
| 6 | logCHIMP_det ~ -1 + Intercept + cultivated_fruit + wild_fruit + dist_road + dist_forest + dist_agriculture + dist_village + hum_det | -734.9826 |
| 7 | logCHIMP_det ~ -1 + Intercept + mango_baobab + orange_lime_papaya + wild_fruit + dist_road + dist_agriculture + dist_village | -734.7137 |
| 8 | logCHIMP_det ~ -1 + Intercept + mango_baobab + wild_fruit + dist_road + dist_village + dist_agriculture | -734.3467 |
| 9 | logCHIMP_det ~ -1 + Intercept + cashew + mango_baobab + orange_lime_papaya + palm_fruit + ten_wild_fruits + dist_forest + dist_road + dist_agriculture + dist_village + hum_det | -734.089 |
| 10 | logCHIMP_det ~ -1 + Intercept + cashew + mango_baobab + palm_fruit + ten_wild_fruits + dist_forest + dist_road + dist_agriculture + dist_village | -732.6589 |
| 11 | logCHIMP_det ~ -1 + Intercept + mango_baobab + orange_lime_papaya + wild_fruit + dist_road + dist_agriculture + dist_village + hum_det | -732.6513 |
| 12 | logCHIMP_det ~ -1 + Intercept + mango_baobab + orange_lime_papaya + wild_fruit + dist_forest + dist_road + dist_agriculture + dist_village | -731.8472 |
| 13 | logCHIMP_det ~ -1 + Intercept + cashew + mango_baobab + palm_fruit + ten_wild_fruits + dist_road + dist_agriculture + dist_village | -731.6167 |
| 14 | logCHIMP_det ~ -1 + Intercept + mango_baobab + orange_lime_papaya + palm_fruit + dist_road + dist_agriculture + dist_village | -731.5851 |
| 15 | logCHIMP_det ~ -1 + Intercept + palm_fruit + dist_road + dist_village + dist_agriculture | -731.1524 |
| 16 | logCHIMP_det ~ -1 + Intercept + mango_baobab + orange_lime_papaya + palm_fruit + dist_road + dist_agriculture + dist_village + hum_det | -731.0261 |
| 17 | logCHIMP_det ~ -1 + Intercept + cashew + mango_baobab + orange_lime_papaya + palm_fruit + ten_wild_fruits + dist_road + dist_agriculture + dist_village | -730.6422 |
| 18 | logCHIMP_det ~ -1 + Intercept + wild_fruit + dist_road + dist_village | -726.5014 |
| 19 | logCHIMP_det ~ -1 + Intercept + palm_fruit + dist_road + dist_village | -723.886 |
| 20 | logCHIMP_det ~ -1 + Intercept | -683.9679 |

**Table S6.** Posterior estimates of hyperparameters of the selected chimpanzee spatiotemporal model showing means, standard deviations and credible interval quantiles.

| Parameter | Mean | ±SD | 2.5% | 50% | 97.5% |
| --- | --- | --- | --- | --- | --- |
| *σ* ^2^*_ε_* | 0.0060 | 0.0017 | 0.0032 | 0.0059 | 0.0099 |
| *σ* ^2^*_ω_* | 0.0408 | 0.0119 | 0.0231 | 0.0387 | 0.0695 |
| *a* | 0.7567 | 0.0491 | 0.6436 | 0.7630 | 0.8356 |
| *ρ* | 582.28 | 149.92 | 330.14 | 568.91 | 914.23 |

$\sigma_{\varepsilon}^{2}$ = measurement error of variance referred to as nugget effect^10^; $\sigma_{\omega}^{2}$ = marginal variance of the Gaussian Markov Random Field^6^; *a* = temporal autocorrelation coefficient AR(1); *ρ* = empirically derived spatial autocorrelation.

Legend for additional supplementary material

Movie S1 (separate file). Predicted chimpanzee spatiotemporal range use across the year, showing intensity of space use increasing at Cadique Nalu village when cultivated fruit were available and wild fruit were scarce. Prediction raster layers were created using the R package RASTER version 3.4-5^16^. Figures were created using QGIS (https://www.qgis.org/en/site/). Bottom graphs show the temporal availability of village-only cultivated ripe fruit (left) and that of eleven wild ripe fruit important to chimpanzees (right).

**Supplementary Information References**

1. Hockings, K. J., Parathian, H., Bessa, J. & Frazão-Moreira, A. Extensive overlap in the selection of wild fruits by chimpanzees and humans: implications for the management of complex social-ecological systems. *Front. Ecol. Evol.* **8**, (2020).

2. INE. *Recenseamento geral da população e habitação: população por região, sector e localidades por sexo censo 2009*. 160 (2009).

3. Hockings, K. J. & Sousa, C. Differential utilization of cashew—a low-conflict crop—by sympatric humans and chimpanzees. *Oryx* **46**, 375–381 (2012).

4. Costa, S., Casanova, C. & Lee, P. What does conservation mean for women? the case of the Cantanhez Forest National Park. *Conserv. Soc.* **15**, 168–178 (2017).

5. Abrahamsens, P. *A review of Gaussian random fields and correlation functions*. 70 http://publications.nr.no/917Rapport.pdf (1997).

6. Lindgren, F., Rue, H. & Lindström, J. An explicit link between Gaussian fields and Gaussian Markov random fields: the stochastic partial differential equation approach. *J. R. Stat. Soc. Ser. B Stat. Methodol.* **73**, 423–498 (2011).

7. Blangiardo, M. & Cameletti, M. *Spatial and spatio-temporal Bayesian models with R - INLA*. (John Wiley & Sons, 2015).

8. Blangiardo, M., Cameletti, M., Baio, G. & Rue, H. Spatial and spatio-temporal models with R-INLA. *Spat. Spatio-Temporal Epidemiol.* **4**, 33–49 (2013).

9. Cameletti, M., Lindgren, F., Simpson, D. & Rue, H. Spatio-temporal modeling of particulate matter concentration through the SPDE approach. *AStA Adv. Stat. Anal.* **97**, 109–131 (2013).

10. Cressie, N. & Wikle, C. K. *Statistics for spatio-temporal data*. (John Wiley & Sons, 2015).

11. Lindgren, F. & Rue, H. Bayesian spatial modelling with R-INLA. *J. Stat. Softw.* **63**, 1–25 (2015).

12. Bessa, J., Sousa, C. & Hockings, K. J. Feeding ecology of chimpanzees (*Pan troglodytes verus*) inhabiting a forest-mangrove-savanna-agricultural matrix at Caiquene-Cadique, Cantanhez National Park, Guinea-Bissau. *Am. J. Primatol.* **77**, 651–665 (2015).

13. McLennan, M. R. Chimpanzee ecology and interactions with people in an unprotected human-dominated landscape at Bulindi, Western Uganda. (Oxford Brookes University, 2010).

14. Sun, C. *et al.* Tree phenology in a tropical montane forest in Rwanda. *Biotropica* **28**, 668–681 (1996).

15. Saw, L. G., LaFrankie, J. V., Kochummen, K. M. & Yap, S. K. Fruit Trees in a Malaysian Rain Forest. *Econ. Bot.* **45**, 120–136 (1991).

16. Hijmans, R. J. *Raster: geographic data analysis and modeling.* (2020).
